# Diagnosing Gender Bias in Image Recognition Systems

**DOI:** 10.1177/2378023120967171

**Published:** 2020-11-11

**Authors:** Carsten Schwemmer, Carly Knight, Emily D. Bello-Pardo, Stan Oklobdzija, Martijn Schoonvelde, Jeffrey W. Lockhart

**Affiliations:** 1 GESIS–Leibniz Institute for the Social Sciences, Cologne, Germany; 2 New York University, New York, NY, USA; 3 American University, Washington, DC, USA; 4 California YIMBY, Sacramento, CA, USA; 5 University College Dublin, Dublin, Ireland; 6 University of Michigan, Ann Arbor, MI, USA

**Keywords:** gender, image recognition, computational social science, bias, stereotypes

## Abstract

Image recognition systems offer the promise to learn from images at scale without requiring expert knowledge. However, past research suggests that machine learning systems often produce biased output. In this article, we evaluate potential gender biases of commercial image recognition platforms using photographs of U.S. members of Congress and a large number of Twitter images posted by these politicians. Our crowdsourced validation shows that commercial image recognition systems can produce labels that are correct and biased at the same time as they selectively report a subset of many possible true labels. We find that images of women received three times more annotations related to physical appearance. Moreover, women in images are recognized at substantially lower rates in comparison with men. We discuss how encoded biases such as these affect the visibility of women, reinforce harmful gender stereotypes, and limit the validity of the insights that can be gathered from such data.

Bias in the visual representation of women and men has been endemic throughout the history of media, journalism, and advertising (Becker 1974; Ferree and Hall 1990; Goffman 1967). As Goffman (1976:11) argued, such “public pictures” are a key symbolic arena in which gendered “social structure of hierarchy or value” is manifested and reproduced. Yet despite their importance, social science research has largely neglected the analysis of images as an arena of social and political valuation. Until recently, the complexity of images rendered large-scale, systemic analysis a near impossibility.

The advent of automated image labeling and recognition systems has increased the importance of images as a form of social data, facilitating their widespread use in commercial enterprise (e.g., Greenfield 2018; HG Insights 2020) and, increasingly, for social research (e.g., Di Ronco and Allen-Robertson 2020; Garimella and Eckles 2020; Geboers and Van de Wiele 2020; Gelman, Mattson, and Simpson 2018; Webb Williams, Casas, and Wilkerson 2020; Xi et al. 2019). At the same time, recent research has shown algorithmic classification systems to be mechanisms for the reproduction, and even amplification, of more general social biases (Friedman and Nissenbaum 1996; Noble 2018). Thus far, several recent studies have detailed gender biases affecting supervised image recognition systems. For example, image search algorithms, when asked to return images for occupations, generated results that reproduced gendered stereotypes, exaggerating gender disparities (Kay, Matuszek, and Munson 2015) and featuring women less prominently than men (Lam et al. 2018).

Although these studies have shown how image recognition systems produce bias in the representation of women and men (i.e., how many appear in photos), less research has systematically explored bias in the content of these algorithms’ results (i.e., how images of women and men are differently labeled, tagged, and categorized). In this article, we present an analysis of bias in both the identification of people and the content labeling of images of women and men across a set of popular commercial image recognition systems. To the best of our knowledge, this article is the first to systematically evaluate biases across both these dimensions of person identification and content labeling. We draw upon data from a particularly salient social arena: the visual communications of American politicians. Using two data sets of images from members of the 115th Congress, we analyze how Google Cloud Vision (GCV)—a widely used service in industry and scientific research—categorizes these politicians’ images. We replicate our analysis across other popular off-the-shelf alternatives, including Microsoft Azure Computer Vision and Amazon Rekognition. Across both data sets and all three platforms, we find consistent evidence of two distinct types of algorithmic gender bias. Image search algorithms not only exhibit bias in identification—algorithms “see” men and women and different rates—but bias in content, assigning high-powered female politicians labels related to lower social status.

Following studies of gender, classification, and status inequalities (Ridgeway 2011, 2014; Ridgeway and Correll 2004) we suggest that image recognition systems reproduce the status inequalities and gender stereotypes at play in wider social structure. These algorithms not only lead to differences in the representation of men and women but systematically categorize women and men with labels differentiated by status. Empirically, we conclude that the systematic nature of such biases in image recognition classifiers renders these classifiers unsuitable for gender-related analyses. The pervasive and not always obvious nature of these biases means they may also confound analyses that are not gender focused. Theoretically, our findings identify these algorithms as an important case of what Ridgeway (2011:40) termed an “amplification process,” that is, a mechanism through which gender differentials are reinscribed into novel social arenas and social forms.

## Gender Inequality, Categorization, and Algorithmic Bias

Gender inequality is characterized by, and reproduced through, the persistence of gendered stereotypes that associate women with lower social status than men (Eagly, Wood, and Diekman 2000; Ridgeway 2011, 2014; Ridgeway and Correll 2004). As Ridgeway (2011:11) argued, gender is “at root a status inequality,” one based on cultural beliefs about the differential hierarchical status between men and women. Widely held and enduring gender beliefs characterize women as less agentic, less worthy, and less competent than men (Conway, Pizzamiglio, and Mount 1996; Fiske et al. 2002; Lueptow, Garovich-Szabo, and Lueptow 2001; Spence and Buckner 2000). Whereas women are typically associated with “communal tasks,” men are typically seen as “more competent at the things that ‘count most’” and that earn the highest esteem (Ridgeway and Correll 2004). These same stereotypes have been shown to be at play in the visual representation of men and women (Ferree and Hall 1990; Goffman 1976). For instance, in *Gender Advertisements*, Goffman (1976) demonstrated how advertisements systematically portrayed women in an “unserious,” childlike fashion. Ferree and Hall (1990) found that even in sociology textbooks, a corpus supposedly attentive to gender inequalities, images reflected women’s marginality in the domains of politics and the economy.

A great deal of social science research has investigated the puzzling endurance of these gender stereotypes over time (Cotter, Hermsen and Vanneman 2011; England 2010; Lueptow, Garovich-Szabo, and Lueptow 2001): beliefs that are continually reinscribed “in new social forms of social and economic organization as these forms emerge in society” (Ridgeway 2011:4). A key mechanism for this persistence is the ability of gendered status beliefs to “transfer” to novel social arenas, what Ridgeway termed an “amplification process.” This amplification process allows categorical differences associated with gender to expand in their range of application, so that preexisting gender beliefs are carried into new industries, occupations, or social forms. Status beliefs can even be transferred to “non-status elements” (Tak, Correll, and Soule 2019). For example, gendered stereotypes about men and women can transfer to evaluations of the products they produce, with women being disadvantaged when they produce stereotypically male-typed goods (Tak et al. 2019).

This research has typically focused on how status inequalities are perpetuated through gender beliefs: individuals bring either conscious or subconscious gendered classifications to novel social arenas (Correll and Ridgeway 2003; Webster and Foschi 1988). The promise of machine-learning algorithms has been that they would bypass this aspect of human bias, leading to more accurate or equitable results (Cowgill 2018; Gates, Perry, and Zorn 2002; Kleinberg, Ludwig et al. 2018). Nevertheless, a growing body of research has shown that algorithms propagate, and even amplify, existing social structures and biases (Angwin et al. 2016; Benjamin 2019; Noble 2018; Sandvig et al. 2016). That is, algorithms are “not cameras onto social realities but engines” (Fourcade and Healy 2017), reproducing preexisting categorizations found in the social institutions from which the algorithm emerges. For example, natural language processing trained on biased text has been shown to strengthen the gendered associations in language, rather than avoiding them (Benjamin 2019; Bolukbasi et al. 2016; Noble 2018).

Although more research has been conducted on text than images, prior studies of images have shown similar patterns (Buolamwini and Gebru 2018). Some scholars, many of them computer scientists, have begun to analyze what Ferree and Hall (1990:505) referred to as the “first level of representation” in image bias: estimating the systemic absence of images of women in particular social arenas. For example, in a study of hundreds of thousands of news articles, Jia, Lansdall-Welfare, and Cristianini (2015) found that the representation of women varied by topic, with political images featuring primarily men. This bias in representation can then be encoded into biases in algorithms. For example, in a study of occupations, Kay et al. (2015) found that search engine algorithms returned images that overrepresented men compared with their actual numbers in the population.

To date, less research has investigated how image-labeling algorithms categorize, that is, how they classify, label, and annotate images of women and men. As Ferree and Hall (1990) suggested and Noble (2018) found, the lower social status of women could result in visual portrayals of women associated with “demeaning or marginalized social positions” (Ferree and Hall 1990:506). The capacity for algorithms to amplify these preexisting biases is the subject to which we now turn.

### From Bias in the World to Bias in the Algorithm

Images are a powerful medium of communication. They are more likely to be remembered than words (Grady et al. 1998; Whitehouse, Maybery, and Durkin 2006) and evoke stronger emotions (Brader 2005) and higher levels of social engagement than text (Rogers 2014). Despite the enormous social scientific potential of images as data, their analysis remains computationally demanding. Algorithms to analyze images often require a high level of technical training and knowledge to design and use, as well as large amounts of training data and data labels. Gathering tens of thousands or more images, all with labels describing their content, remains both costly and time consuming (Chen et al. 2015).

**Figure 1.**
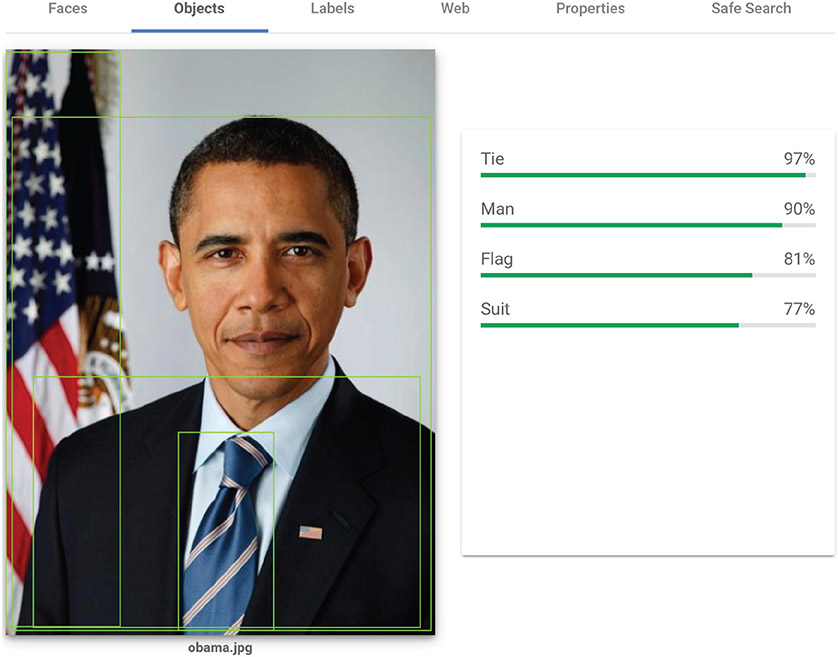
Example of the information that Google’s Cloud Vision platform can return when asked to label a portrait of former U.S. president Barack H. Obama.

Commercial image labeling services, available to the public from Google, Amazon, Microsoft, and other companies since 2016, provide an alternative to this onerous process: reducing the cost of labeling images and identifying their content at scale and offering the potential to make image analysis readily available to users not trained in designing neural networks. These platforms allow users to quickly and easily retrieve labels for any image, as shown in Figure 1. A recent study shows just how drastic is the difference in effort between human coders and algorithms such as GCV: “the API codified 1,818 images in less than 5 min, whereas the human coder spent nearly 35 hours to complete the same task” (Bosch, Revilla, and Paura 2019).

One widely known dimension of systems such as GCV that rely on machine learning is that they seek out and then reproduce patterns in the data on which they are trained. Input data are typically “found data” from the “real world,” containing the biases and cultural associations of human societies, which then get reproduced as “objective” and “scientific” decisions from algorithms (Benjamin 2019). For example, ImageNet is a database widely used to train image-labeling algorithms that maps the categories from Princeton’s WordNet to more than 14 million images scraped from the Internet (Crawford and Paglen 2019). WordNet is a taxonomy of English terms dating to the 1980s, based on pre-1972 Library of Congress taxonomies, that contains numerous racist, ableist, and misogynistic terms (Crawford and Paglen 2019). When ImageNet’s designers and human coders linked these terms to pictures of people from the Internet, they encoded those biases into the database. As Crawford and Paglen (2019) showed, this profoundly shaped algorithms that were trained using the database. After their work, ImageNet removed many of the most offensive labels (Ruiz 2019).

Input data are not the only social influence on algorithmic systems. Computer engineers’ design decisions and tweaking of automated systems also encode biases (Seaver 2018). For example, engineers working on music playlist algorithms not only employed users’ behavior to code their algorithms but also personally listened to the playlists they generated, tweaking the way the algorithms used their input data until the engineers thought the output sounded good (Seaver 2018). As Seaver stated, arbitrary preferences and biases outside the code therefore became a part of the algorithm:

The essence of a contemporary algorithmic system [is] a steady accumulation of feedback loops, little circuits of interpretation and decision knit together into a vast textile. Every stitch is held together by a moment of human response, a potential rejection shaped by something outside the code, whether it is the arbitrariness of personal preference, the torque of structural bias, or the social force of a formal evaluation framework. (p. 377)

Notably, the algorithmic systems trained on these input data are increasingly “black boxes.” A system is a black box either if its technical design is sufficiently complex that human users cannot interpret the meaning of the inner workings or if the details of the system’s design and construction are hidden from users, for example, as corporate trade secrets (Rudin 2019). This second kind of black box describes GCV and nearly every commercially available “algorithm” or scoring system. Only some Google employees know which data sets and design decisions went into building and tuning GCV. Therefore, although researchers can audit the results of algorithms, they generally cannot recover the true process or logic of the black box’s decisions, and attempts to reverse-engineer the decision process “are misleading and often wrong” (Rudin 2019:211).

Thus far, scholars working on images have taken some initial steps to avoid the bias potentially introduced by these algorithms. For instance, in a study on social media images of legislators, Xi et al. (2019) removed all women and members of racial and ethnic minority groups from their data in order to sidestep gender and racial biases. Although such an approach may be reasonable for specific research questions, it should be a last resort: systematically excluding large swaths of the population not only can lead to nongeneralizable inferences, it can also bias social scientific research away from pivotal research questions on inequities in social, political, and economic visual communication (Rossiter 1993). We suggest that prior to resorting to such data limitations, we should develop a better understanding of the systematic nature of such biases. In what follows, we draw upon existing literature to examine gender algorithmic bias across two dimensions: bias in identification and bias in content.

### Two Dimensions of Image Bias

Bias in identification is an analog to what Ferree and Hall termed the “first level of representation”: at a very basic level, does the algorithm *see* people with equal accuracy regardless of their gender? For the most part, this has been the primary focus of the “algorithmic bias” literature, which has defined algorithmic injustice and discrimination as situations where errors disproportionately affect particular social groups (Noble 2018).

Bias in content, by contrast, is possible when algorithms output only a subset of possible labels, even if the output is correct. In this case, an algorithm might systematically return different subsets of correct labels for different groups of people. We formalize this as “conditional demographic parity” (Corbett-Davies et al. 2017). Conditional on image content, an algorithm is considered biased if it returns labels at different rates for different demographic groups. For instance, if men and women in a sample wear suits at equal rates, then an unbiased algorithm would return the label “suit” equally often for each gender. Why might the presence or absence of women in a photo affect the identification of such seemingly nongendered classifications such as clothing items? Algorithms learn by observing associations in the data they are trained on (i.e., data the models are fitted to). If we fit an algorithm to a data set in which all men had suits, and no women did, it might well learn that the probability of “suit” being the right answer, given that it sees a woman or features associated with women like long hair, is extremely low. When later presented with images of women in suits, then, it would be unlikely to label them “suit,” even though that is a correct label.

Input biases do not need to be that extreme to have these effects, however. Research on word embeddings has found that algorithms can pick up far more subtle associations (Kozlowski, Taddy, and Evans 2019). For example, one team found that word2vec trained on Google News articles produced gendered analogies such as “man is to computer programmer as woman is to homemaker.” This is because gender-specific words (such as *sister* or *mother*) may be statistically associated with gender-neutral words (such as *homemaker*) in text, and thus algorithms that attempt to identify meaning through observed associations amplify these biases (Bolukbasi et al. 2016). Similarly, algorithms trained on real-world images may convert associations between gender-specific labels and gender-neutral labels into biased results for image content.

## Data

To identify bias in identification and content in image recognition systems, we use two data sets containing images associated with members of the 115th U.S. Congress: a data set of official headshots and a set of images tweeted by these members. We have several reasons for focusing our analysis on political images. First, politicians’ image use is substantively important. The political realm has consistently revealed gender bias in the representation of women in images (Jia et al. 2015). It is important to know whether and how human bias in the production and use of images plays out in algorithmic labeling of images. To date, politics has been an important domain of social science research on images (e.g. Anastasopoulos et al. 2016; Casas and Webb Williams 2019; Webb Williams et al. 2020; Xi et al. 2019).

Second, our data sets offered a unique opportunity to study the bias in black-boxed image classification algorithms. We compiled two matched data sets: (1) a control data set consisting of uniform portraits of the members of Congress (MCs) themselves and (2) a found data set of images these politicians tweeted. The control data set limits the variation in image content and style, making it easier to detect biases in algorithmic performance, while remaining a real-world image data set. It includes social markers of gender, age, race, and politics such as clothing, hair, jewelry, and flags that are essential to sociological understandings of identity and appearance but that are typically cropped or abstracted away in the controlled photographs of laboratory studies. The found data set is composed of images shared by the politicians’ official Twitter accounts, which are highly variable in content, style, and purpose but which still share a general context. These characteristics mirror those of many digital sociology and archival research projects, allowing us to evaluate algorithmic bias in a setting relevant to other researchers. Both data sets are linked to the same set of politicians, and thus the same demographics, enabling us to compare findings.

### Control Data Set

We acquired the control data set by extracting official portraits of MCs from Wikipedia. These photos are produced by the U.S. Government Printing Office for the official *Congressional Pictorial Directory*, which contains photos and biographical details for all MCs during a given session. The vast majority of these images are taken in front of a neutral monochrome background. In many photos, an American flag is positioned to the MC’s right, and in a subset of those photos, the flag of the MC’s home state is also displayed to that person’s left. Many photos are taken either somewhere in the U.S. Capitol or an MC’s office. In every photo, the vast majority of the frame is occupied by the MC. Similarly, in all photos, MC’s are clothed in civilian business attire and looking at the camera. MCs all have the same occupation, nationality, and motivation for taking their portraits. These photographs are as homogeneous as any real-world set of images might be, without artificially removing socially meaningful aspects of age, gender, race, and ethnicity, such as hair and clothing, which are often removed in laboratory facial recognition data sets. All images fall under the public domain and are included in our replication material. We merged these photos with information about the MCs from government Web sites as well as a public GitHub repository (United States Project 2020).

### Found Data Set

Our found data set is composed of images posted on Twitter by MCs between January 2017 (the start of the 115th Congress) and June 2018 (*n* = 198,170). We obtained the set of images by using the Twitter application programming interface to download each MC’s timeline, limited to his or her most recent 3,200 tweets because of data restrictions from the application programming interface. We then downloaded all of the images these tweets contained.

From these sets of images, we selected a weighted sample in order to validate GCV’s labels with humans’ labeling (*n* = 9,250). An image’s weight for sampling is calculated using both the labels from GCV and the characteristics of the MC posting the image. Image weights are inversely proportional to how rare their features are, such that images with uncommon labels and coming from MCs from underrepresented groups are more likely to be sampled. More details on our sampling strategy are available in the Online Appendix. On average, GCV returned 5.3 labels per image, and we selected only labels to which GCV assigned ≥0.75 confidence (confidence scores from GCV vary between 0.5 and 1.0). In that sense, our validation sample can be regarded as conservative; we evaluate only labels GCV considers highly likely to be applicable to the specific image.

## Methods

Our main analysis is conducted on Google Cloud Vision (GCV). As discussed above, GCV is widely used in industry, and unlike its primary competitors, Amazon Rekognition and Microsoft Azure Computer Vision, GCV shares its underlying technology with the world’s largest Internet image search platform (Google Image Search) and other ubiquitous services such as Google Photos (integrated with every Android phone). We also provide brief analysis of both other platforms showing that our findings generalize outside of GCV.

### Validation

To validate the image labels produced by the algorithm, we hired workers through Amazon’s Mechanical Turk (MTurk). This service has become popular with researchers in several disciplines over the past decade and allows hiring a readily accessible and diverse population of research assistants. Although “MTurkers” have often been a population sampled for survey research (Huff and Tingley 2015), these workers have also been employed to assist in the research process itself (Shank 2016), as was the case for our project. The use of temporary and anonymous workers who lack the labor protections of traditional research assistants employed through a higher education institution has been discussed extensively by other scholars (see Pittman and Sheehan 2016; Williamson 2016). Aspiring to maintain ethical research practices, we paid MTurkers working on our project a “living wage” of $15/hour, more than twice the U.S. federal minimum wage at the time of writing.

We presented each worker with 30 images and a set of five potential labels for each. Some labels were assigned by GCV for corresponding images (positive labels); others were chosen at random from the set of GCV labels assigned to other images but not to the one at hand (negative labels). Each image was coded by at least three people.

Workers were presented with an image and two questions. The first question presented all labels in random order and asked workers to select all labels that applied to the image they were seeing. The second question asked workers to indicate if they saw any men, women, children, or none in the image. Each person validated the labels of 30 images, and multiple people saw each combination of labels and images. Overall, respondents had an agreement rate of 0.77 with one another.

To identify bias in identification, we evaluate whether GCV recognizes men and women in images. With our control data, we have ground truth about the presence and gender of MCs depicted. With our found data, we do not know the true gender of people in images. Instead, we compare whether GCV recognizes men, women, both, or neither in an image to whether human coders do. Human coders and GCV both rely on the same visual gender cues, so our research design measures whether those cues influence the algorithm’s person identification.

Bias in content requires a slightly different approach. There are many things that could be labeled in any image (“an image is worth a thousand words”), but image labeling systems typically return only a handful of labels (an average of 5.3 per image in our data). Even if labels a system returns are correct, it is possible to have bias in which subset of possible correct labels gets returned for a given image. Thus, we measure bias in content as conditional demographic disparity: conditional on actual image contents, we examine whether some labels are disproportionately applied to images containing one demographic group or another.

To measure bias, we rely on two procedures. First, we use χ^2^ test statistics with Yates’s correction on labels to identify which labels are identified relatively most often in portraits of and images tweeted by women compared with men (see the Online Appendix). Second, we use negative binomial regressions to obtain the expected counts of GCV labels in each of five coded categories for the MCs. A negative binomial distribution allows us to model counts while correcting for overdispersion.

Finally, we include several controls. Because recent research suggests that GCV results may depend upon race and skin tone (Noble 2018), we control for race (coded as white or nonwhite). Women are unequally distributed across parties, and to ensure that results are not party dependent, our models also control for party membership (Democrat or Republican). Finally, as studies have shown that the performance of image recognition algorithms may depend upon the age of individuals in the images (Ngan and Grother 2014; Michalski 2017), we control for age (see the Online Appendix). Results are robust to the inclusion or exclusion of these controls.

## Detecting Gender Bias in GCV

Bias differs across image classification systems and changes over time. Because of this, researchers using these algorithms will need to do their own evaluations, specific to the tool they are using, the time they are using it, and even the location they are accessing it from. We propose that such evaluations should measure several components. First, as a baseline, researchers should verify the correctness of the labels provided; many applied papers already evaluate this dimension (e.g., Bosch et al. 2019) but because accuracy will be context dependent, such verification is an important first step every time one uses an algorithm. Second, we suggest that researchers identify two forms of algorithmic bias: biases in identification, which is the focus of much “algorithmic bias” literature (e.g., Kleinberg, Ludwig et al. 2018), and biases in content. In what follows, we discuss each of these components drawing on MCs’ use of images on Twitter as a case study, but the procedure we propose is generalizable to other substantive domains.

### Evaluating GCV

The first, most general dimension for evaluating any algorithm is determining the correctness of its results. There are many different measures for evaluating labeling or classification algorithms (Nelson et al. 2018). In general, commercial labeling systems present users with only predicted positive labels (e.g., “there are cats in this photograph”) and not predicted negative labels (e.g., “there are no children”). This can make calculating many measures of correctness difficult. Additionally, calculating measures of correctness requires “ground truth” data about what is “correct.” But users typically turn to labeling algorithms precisely because they do not already have ground truth information about their images.

**Figure 2.**
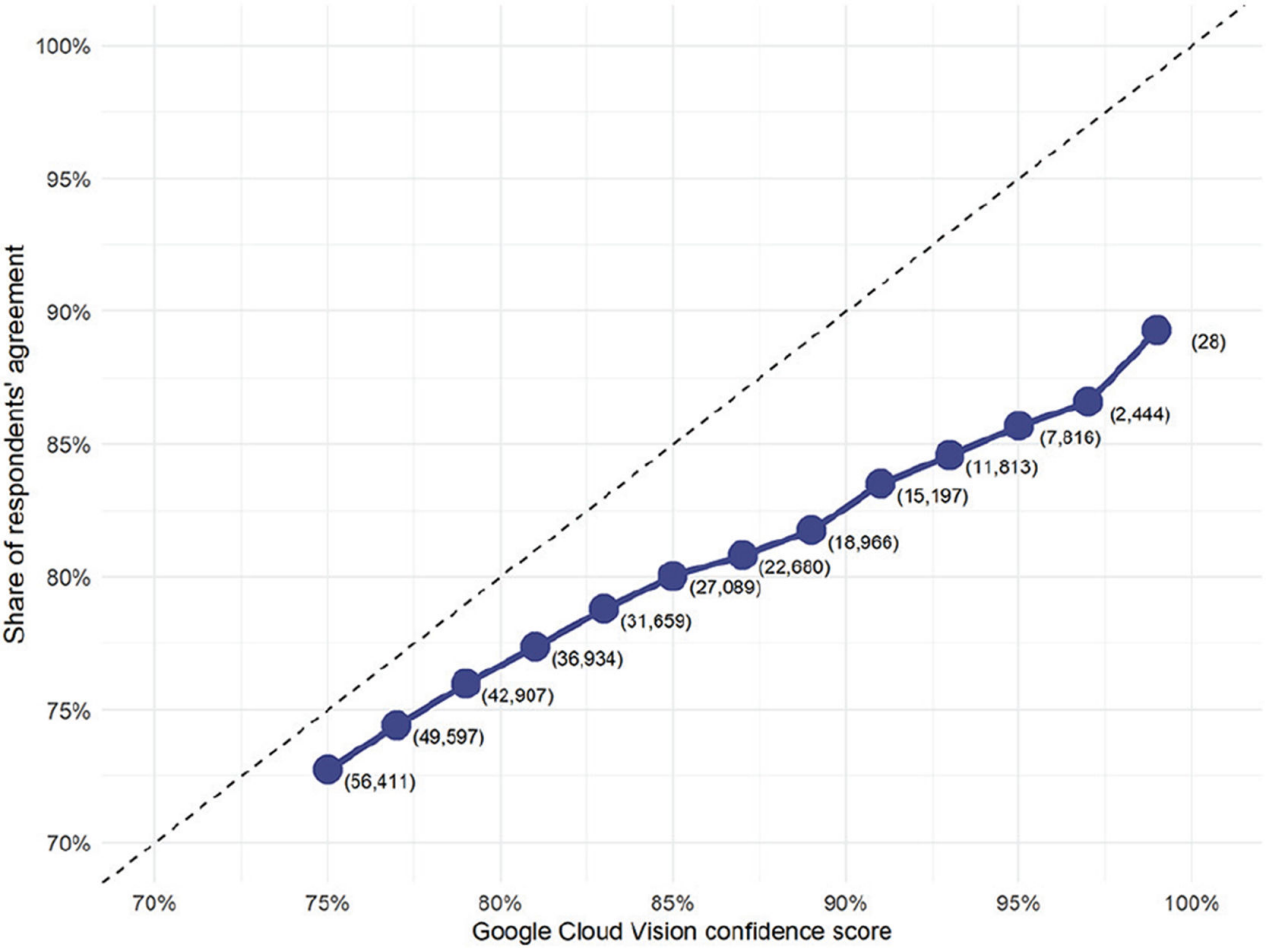
Relationship between Google Cloud Vision (GCV) confidence and human agreement. Numbers in parentheses denote observations for corresponding confidence score thresholds.

We address both challenges using our sample of 9,250 human-coded images. Overall, we find that human crowd workers have high agreement with the labels the GCV algorithm generated, as shown in Figure 2. When presented with an image and a set of potential labels, humans typically select the positive GCV labels, but not the negative labels. Moreover, the proportion of humans who select a label is strongly correlated with the confidence score returned by GCV. That is, GCV’s confidence score is a good measure of whether a human would agree that the label applied to a given image. In this sense, GCV is a high-precision image labeling system: when GCV says that a label applies to an image, it is generally correct.

### Bias in Identification

The overall accuracy of an algorithm such as GCV is not the only important measure, however. As Nelson et al. (2018) showed, sometimes the measures of correctness for individual categories and labels are more important for sociological analysis and can lead to further insights about the data. We test this with gender. Although observer-ascribed gender is a poor measure of gender identity (Hamidi, Scheuerman, and Branham 2018; Lagos 2019), it can be a good measure of the gendered stereotypes about appearance that may influence GCV.

**Figure 3.**
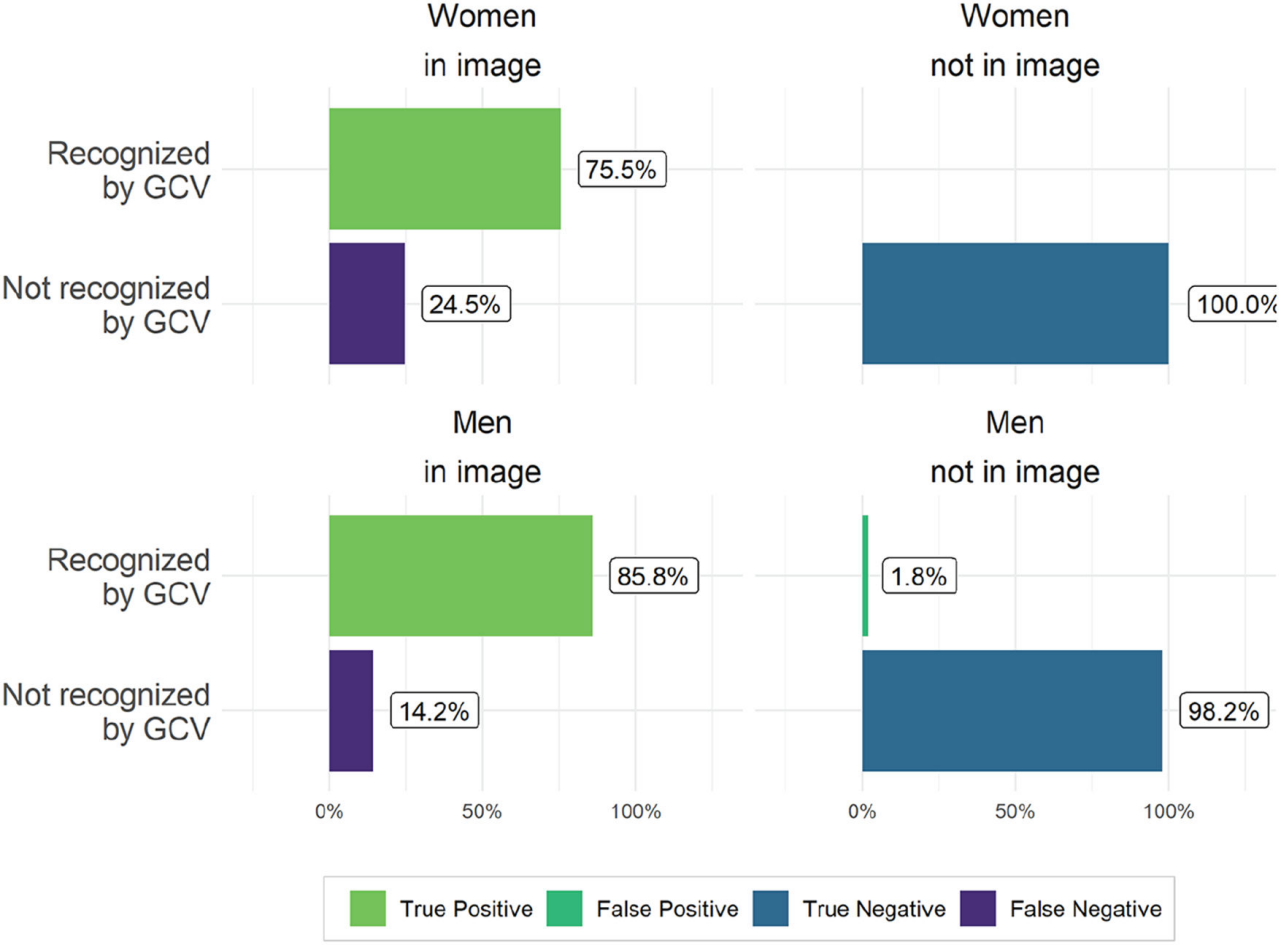
Accuracy of person detection of Google Cloud Vision (GCV). Percentages shown were determined by comparing gender of members of Congress depicted in uniform data (professional photographs) with annotations from the object recognition software.

**Figure 4.**
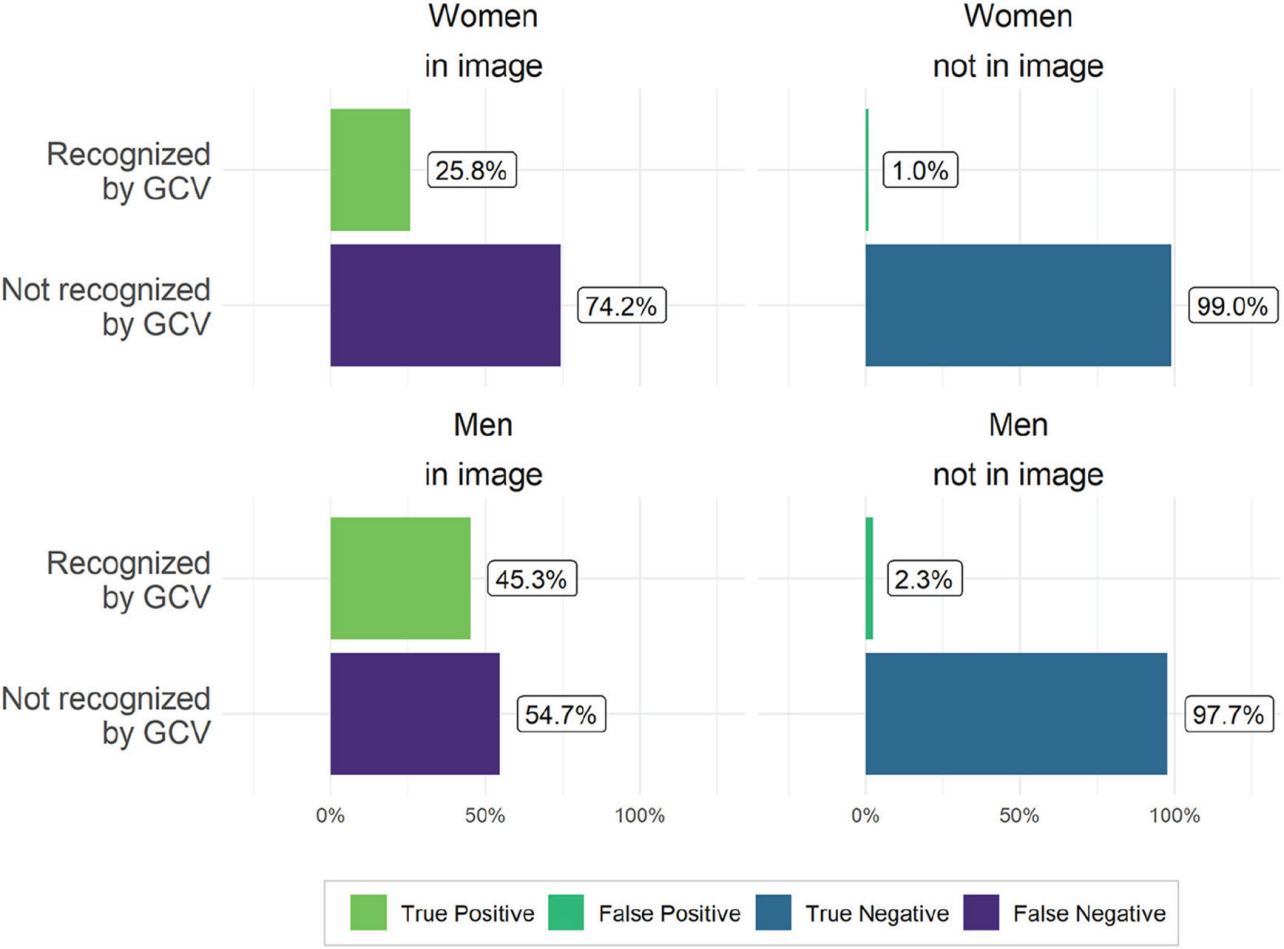
Accuracy of person detection of Google Cloud Vision (GCV). Percentages shown were determined by comparing human agreement about the presence of men or women in Twitter images with annotations from the object recognition software.

We use the object recognition module of GCV which, at the time of data collection, detected people and differentiated between men or women. This feature has since been removed. We conduct this validation using all images from the control data set (results shown in Figure 3) and all 9,250 images from Twitter that human workers coded (results shown in Figure 4). As the right panels of Figures 3 and 4 demonstrate, GCV has low false-positive rates for detecting people whom our human coders did not identify in the images, regardless of gender. The false-positive rate is low for both women (near 0 percent in the Wikipedia image data and about 1 percent in the Twitter image data) and men (1.8 percent in Wikipedia images and 2.3 percent in the Twitter data). In short, GCV rarely detects people in images where humans do not.

However, the algorithm’s false-negative rates vary substantially by ascribed gender. In our control data set of professional portraits of MCs, women in Congress are recognized in only 75.5 percent of images of women in Congress in comparison with 85.8 percent for men in Congress, a difference of 10 percentage points (see the left panels of Figure 3). Thus, in high-quality photos in which only one individual is presented, women are still “seen” by the algorithm significantly less than men.

This difference was even more striking in our primary data set of Twitter photos. Here, GCV identified 45.3 percent of the men that our human coders saw in the pictures but only 25.8 percent of the women, a striking 20 percentage point gap (see the left panels of Figure 4). As with the label annotation results, GCV object labels for people are high precision: if GCV detects a person, it is very likely that humans will agree that there is a person. However, these results indicate that GCV has poor recall: if GCV does not tag something, it may still nevertheless be in the image (ergo the high false-negative rates in recognizing individuals). High precision with low recall is likely an unavoidable feature of labeling images: for any given image, the set of possible correct labels that the algorithm could return is theoretically enormous. Our findings show, however, that there is substantial gender bias in errors of omission: false-negative rates are substantially higher for women than men.

### Biases in Content

The second component of evaluating GCV labels concerns bias in content. Our finding that positive labels are recognized as correct by humans does not rule out bias in their distribution. Positive labels could be both correct and biased, in the sense that they might not always meet conditional demographic parity.

**Figure 5.**
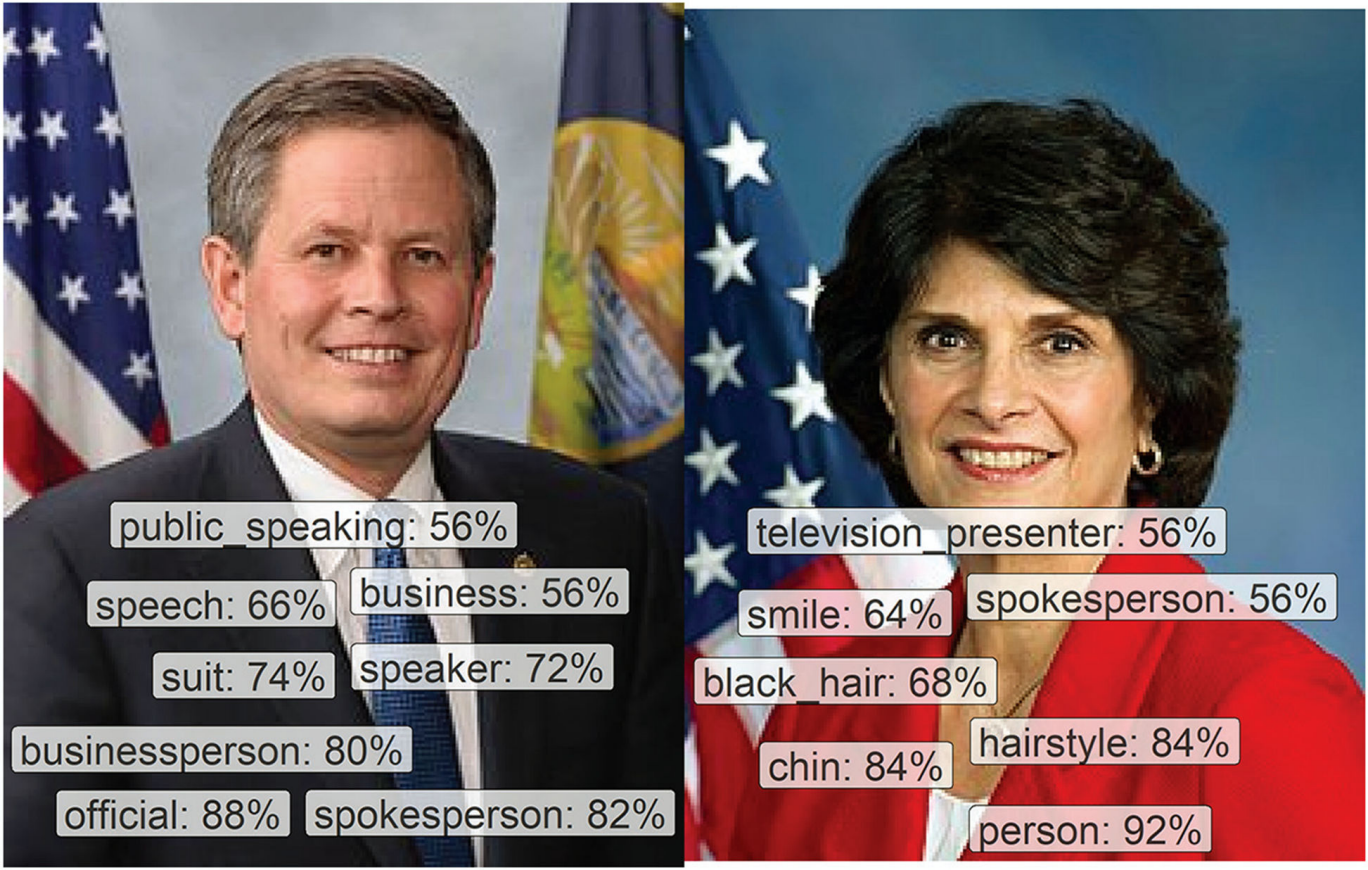
Two images of U.S. members of Congress with their corresponding labels as assigned by Google Cloud Vision. On the left is Steve Daines, a Republican senator from Montana. On the right is Lucille Roybal-Allard, a Democratic representative from California’s 40th congressional district. Percentages next to labels denote confidence scores of Google Cloud Vision.

To examine this possibility, we used GCV labels from our uniform data set of MCs’ professional portraits. If GCV returns gender-biased labels on this set of images, those biases could affect any inferences we draw from the algorithm with other data sets, including our analysis of whether MCs engage in gendered patterns of communication on Twitter. Example images and labels from this set can be seen in Figure 5. Here, GCV labeled Congresswoman Lucille Roybal-Allard as a “smiling” “television presenter” with “black hair,” whereas Senator Steve Daines was labeled as an “official,” “businessperson,” and “spokesperson.”

**Figure 6.**
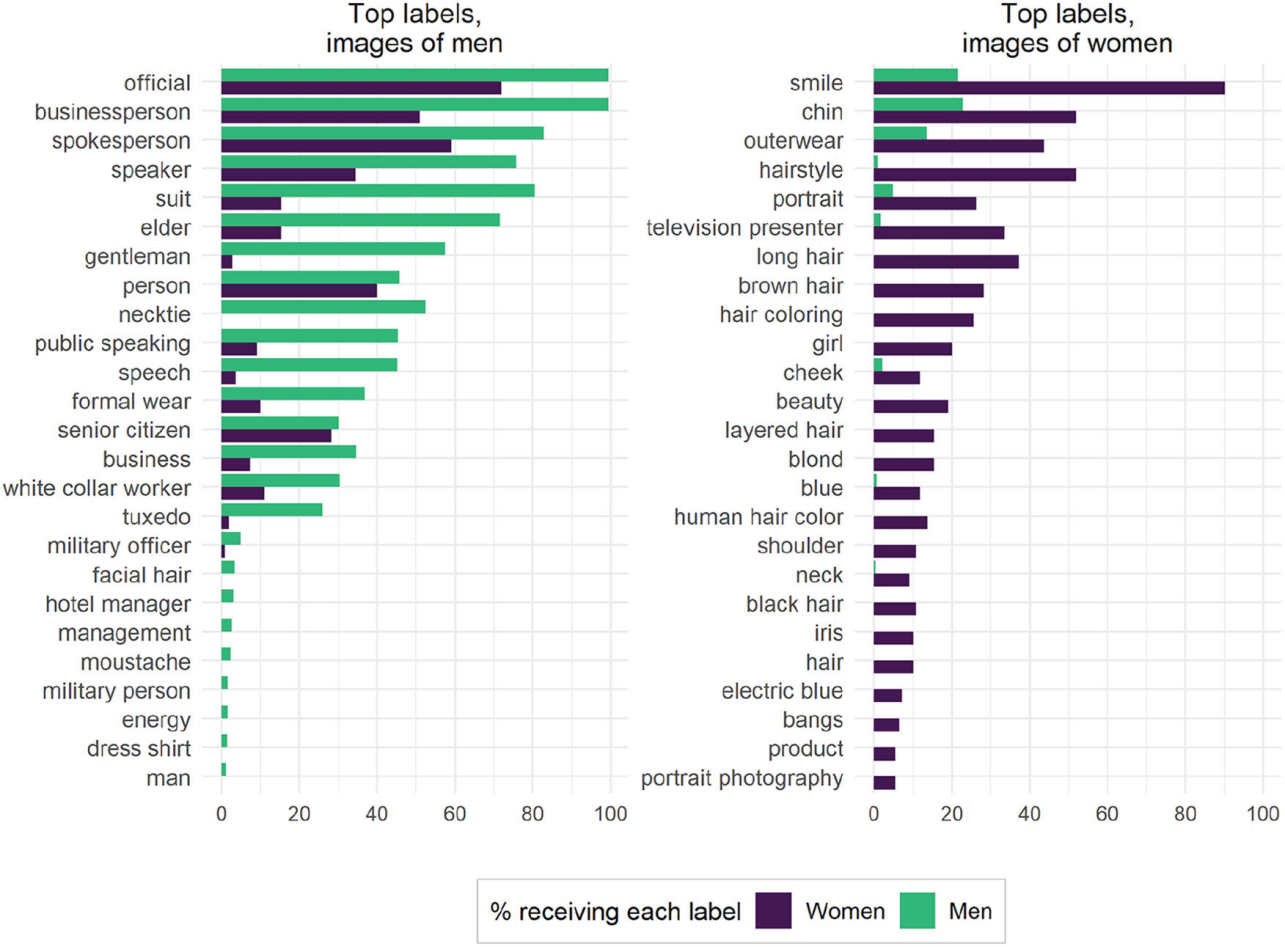
Google Cloud Vision labels applied to control dataset (professional photos). The 25 most gendered labels for men and women were identified with χ^2^ tests (*p* ≤ .01). Labels are sorted by absolute frequencies. Bars denote the percentage of images for a certain label by gender.

We then use χ^2^ tests to identify the key labels by gender for our control dataset (see the Online Appendix for additional information). Figure 6 shows the top 25 key labels for both men and women, sorted by absolute frequencies. Some labels, for instance “long hair” for women, are a clear result of the underlying data we chose: there are no congressmen with long hair in the data set, and no congresswomen who wore neckties, so it is unsurprising that some of these labels have strong gendered associations. Note, however, that “bald” and “short hair” do not appear among the labels GCV returned, indicating a bias in which hairstyles the algorithm mentioned. The seemingly neutral label “hairstyle” is given to more than half of women but only a minute percentage of men. Similar patterns exist for labels such as “black hair” and “brown hair.” By our manual count, 2 percent of women’s portraits have no visible hair (because of hats), 3 percent of men’s portraits have no visible hair (completely bald heads), and a further 7 percent of men’s portraits have partial hair (hair visible on the sides but not the top of the head). Conservatively, then, women are 1.1 times as likely as men in these data to have visible hair, nowhere near the disparity in labels returned by GCV. Thus, we conclude that, conditional on hair being in the image, GCV was much more likely to comment on it if the hair belonged to a woman.

Labels such as “girl” and “gentleman” encode gender directly, so their correspondence with MCs’ gender is unsurprising. However, labeling adult women “girls” while men are labeled with more prestigious and age-appropriate titles such as “gentlemen” is an old, sexist trope (Durepos, McKinlay, and Taylor 2017) that resurfaces in image recognition algorithms.

Furthermore, we see evidence that confirms gender and occupational bias. That is, although all individuals in the data set have the same occupation (MC), GCV labels them with a variety of occupations. Notably, the only occupation with which GCV labels women more often than men is “television presenter,” while men are labeled with more authoritative variants such as “white collar worker,” “spokesperson,” and “military officer.” That is, although these labels are ostensibly gender neutral, their highly gendered cultural histories emerge clearly in GCV’s differential application of the labels. For instance, Perryman and Theiss (2013) showed that the age-diminutive “weather girl” stereotype has developed since the 1950s, when television stations began to hire nonexpert women as presenters to attract viewers through theatrics and sex appeal. Today, GCV labels women as “television presenter” instead of “weather girl,” but the historical gender bias remains evident.

Overall, appearance labels such as “beauty” and “hairstyle” are disproportionately applied to women. Labels most biased toward men revolve around professional and class status such as “gentleman” and “white collar worker.” None of these individual labels is necessarily wrong. Many men in Congress are businesspeople, and many women have brown hair. But the reverse is true as well: women are in business and men have brown hair. From the set of all possible correct labels, GCV systematically selects appearance labels more often for women and high-status occupation labels more for men. Naive analysis using these labels may erroneously conclude that images with men or women in them are more focused on, respectively, business or fashion, even if they are all professional portraits of people with the same occupation.

**Figure 7.**
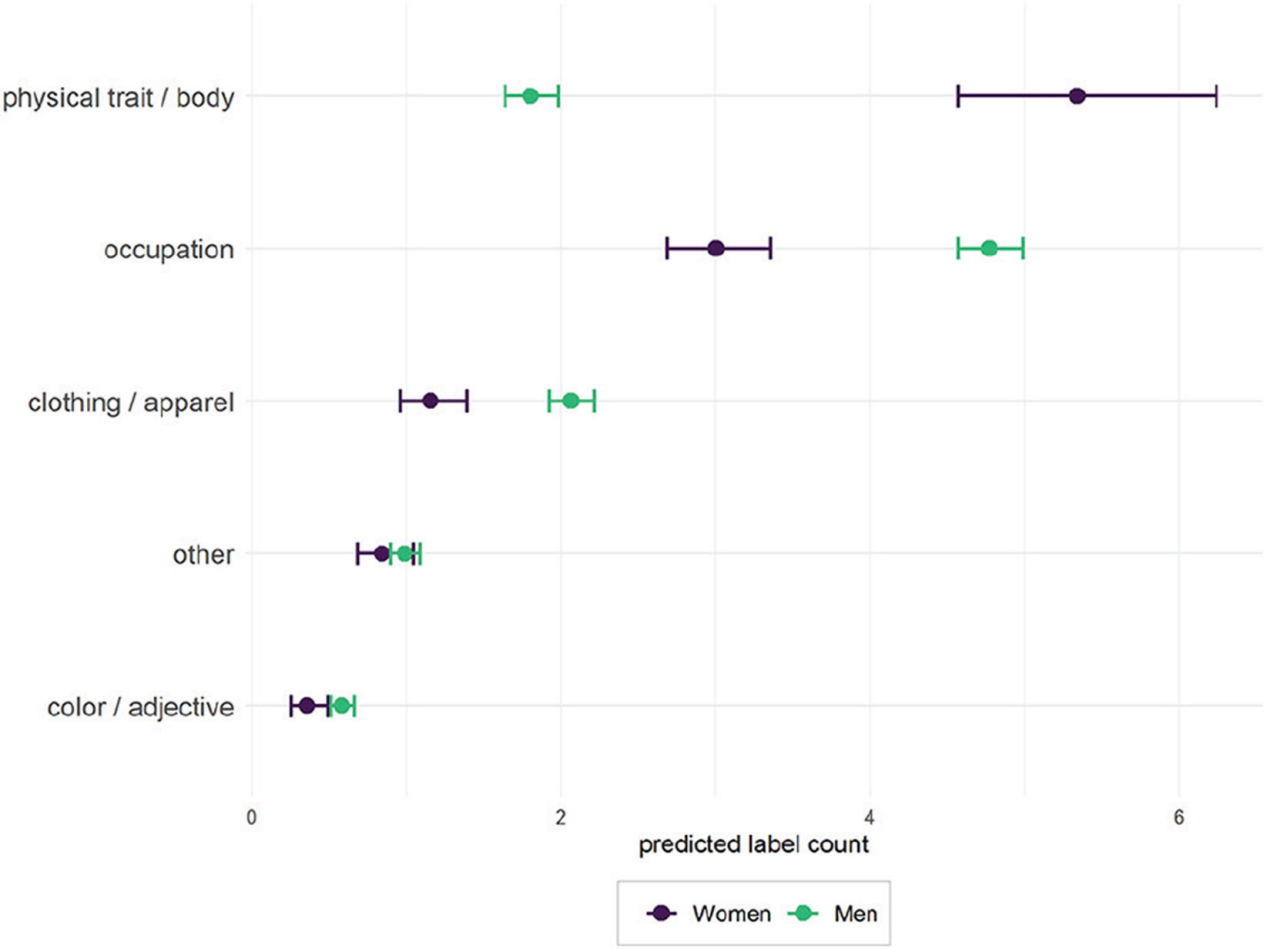
Predicted labels counts for images of men and women. Results are based on the Wikipedia photographs of U.S. members of Congress and negative binomial regressions, controlling for party and ethnicity. Circles describe point estimates, and bars describe 95 percent confidence intervals.

We conducted further analysis to quantify the different types of labels assigned by GCV dependent on gender, race, and party of MCs by manually coding all GCV labels for the photographs of MCs into the following categories: “occupation,” “physical traits & body,” “clothing & apparel,” “color & adjectives,” and “other.” Three authors of this article coded the labels independently, with an intercoder reliability score of 0.88 (see the Online Appendix). For each of these labels, we computed regressions to estimate the effects of gender on label counts for the MC photographs. We opted for negative binomial regressions because dispersion tests for our count-based variables suggested partial overdispersion. We control for race, age, and political party of MCs. Figure 7 shows predictions by gender while holding party, race, and age at observed values.

Images of women receive about 3 times more labels categorized as “physical traits & body” (5.3 for women, 1.8 for men). Images of men receive about 1.5 times more labels categorized as “occupation” (3 for women, 4.7 for men). Images of men also receive more labels related to clothing and apparel than women. We found no substantial differences in labels related to color or adjective or other types of traits.

These results provide further evidence that images of women contain more labels related to physical traits in comparison with images of men. At the same time, labels related to occupation, and to a lesser extent clothing and apparel, are more often included in images of men. Results of the same analysis for ethnicity as well as for political party do not suggest substantial effects (see the Online Appendix). In short, our results indicate that GCV suffers from substantial biases related to gender.

**Figure 8.**
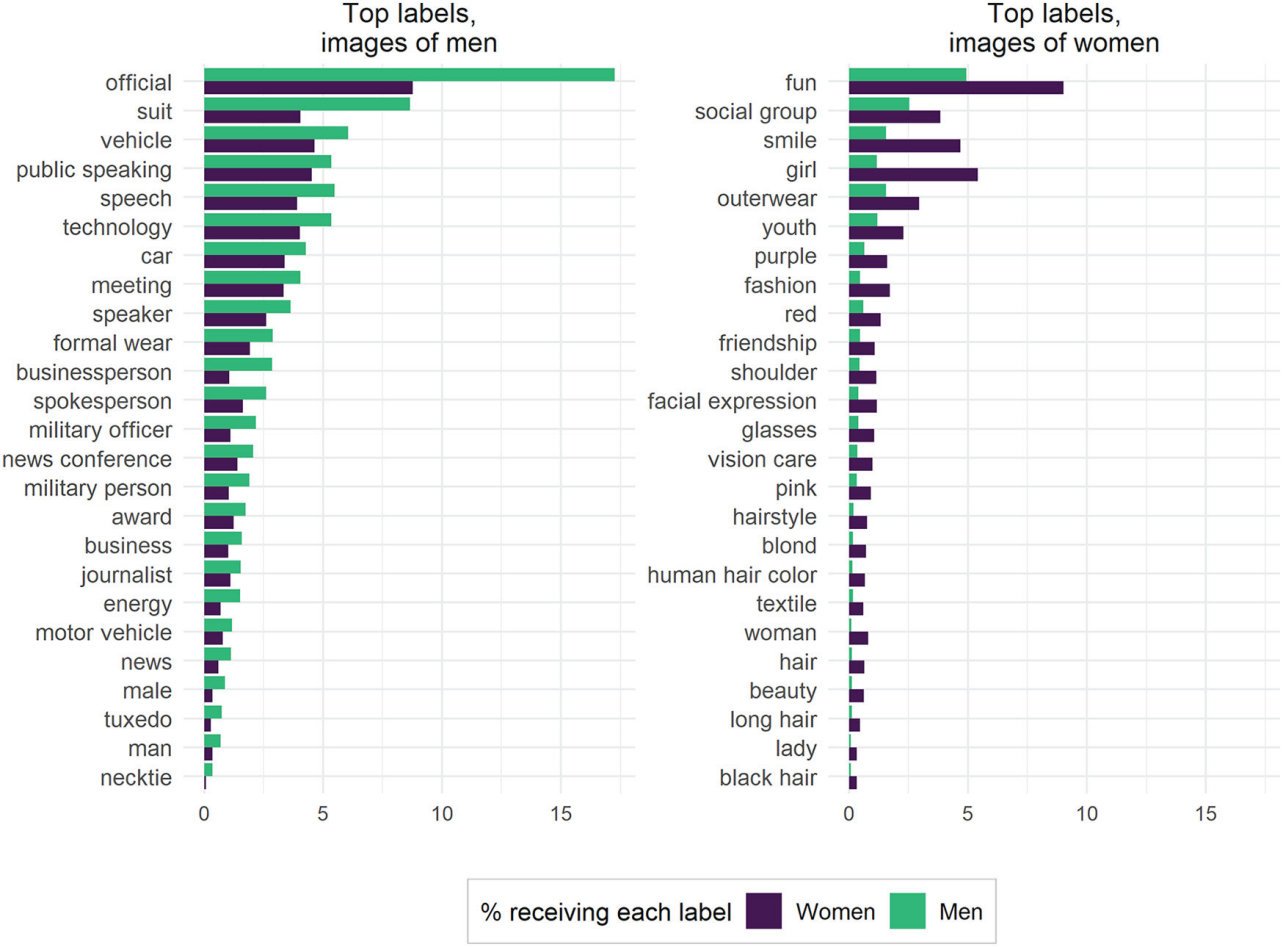
Google Cloud Vision labels applied to found data set (Twitter images). The 25 most gendered labels for men and women were identified using χ^2^ tests (*p* ≤ .01). Labels are sorted by absolute frequencies. Bars denote the percentage of images for a certain label by gender.

To examine how these biases in uniform data manifest in “real world” data, we turn now to our “found” data set of MCs Twitter images. Again, we use χ^2^ tests to identify the labels most strongly associated with images tweeted by male versus female MCs. Figure 8 shows the top 25 key labels for both men and women, sorted by absolute frequencies.

The results indicate a sharp divide in content of images tweeted by men and women, such that women in Congress appear to be much more likely to tweet pictures of women and girls, fashion, and other appearance-focused themes (about 5 percent of all images tweeted by women received the label “girl,” whereas only 1.5 percent of images tweeted by men received that label). Meanwhile, men in Congress appear much more likely to tweet images of officials, vehicles, public speaking, technology, military personnel, and business. These themes conform to common gender stereotypes, and a reasonable but naive interpretation of these results might have been that MCs’ gender substantially influences the content of the images they share on Twitter. The results broken out by MCs’ party affiliations show similarly gendered distinctions (see the Online Appendix).

However, our evaluation procedure highlighted that many of those specific labels are applied with substantial gender bias, which confounds these observed differences. Indeed, when considering that women were much more likely to be given labels associated with physical traits or the body or were much more likely to be labeled as “girls,” many of the most “gendered” findings about images tweeted by MCs are revealed to be artifacts of algorithmic bias. The label “girl,” for instance, does not necessarily indicate the presence of a child, as we identified the biased application of the label “girl” to our control data set of images of adult women. Thus, rather than women tweeting more images of girls than men in Congress, all MCs might simply be tweeting images of themselves that are being labeled differently by GCV.

Our analysis reveals that GCV’s biases severely limit the kind of inferences that scholars interested in gendered political communication could accurately draw from visual evidence if they were to use this black-box algorithm. Indeed, among the top labels associated with “gendered” images tweeted by MCs, it is clear that very few point toward reliable, unbiased differences. We therefore conclude that labels produced by GCV are too biased to yield meaningful insights into gender differences in visual political communication patterns.

### Detecting Bias in Other Image Recognition Tools

**Figure 9.**
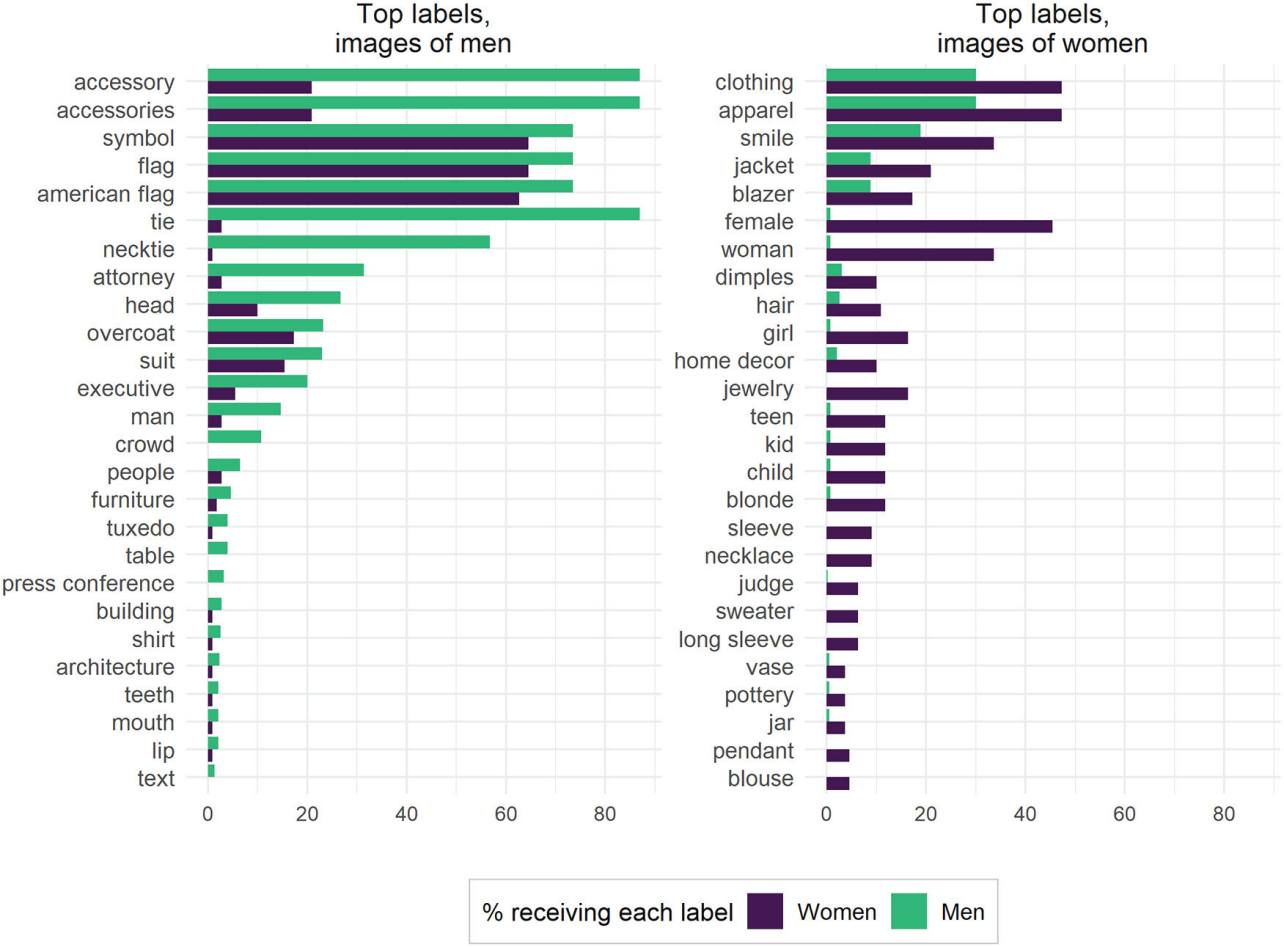
Amazon Rekognition labels applied to professional photographs of members of Congress. The 25 most gendered labels for men and women were identified with χ^2^ tests (*p* ≤ .01). Bars denote the percentage of images for a certain label by gender.

**Figure 10.**
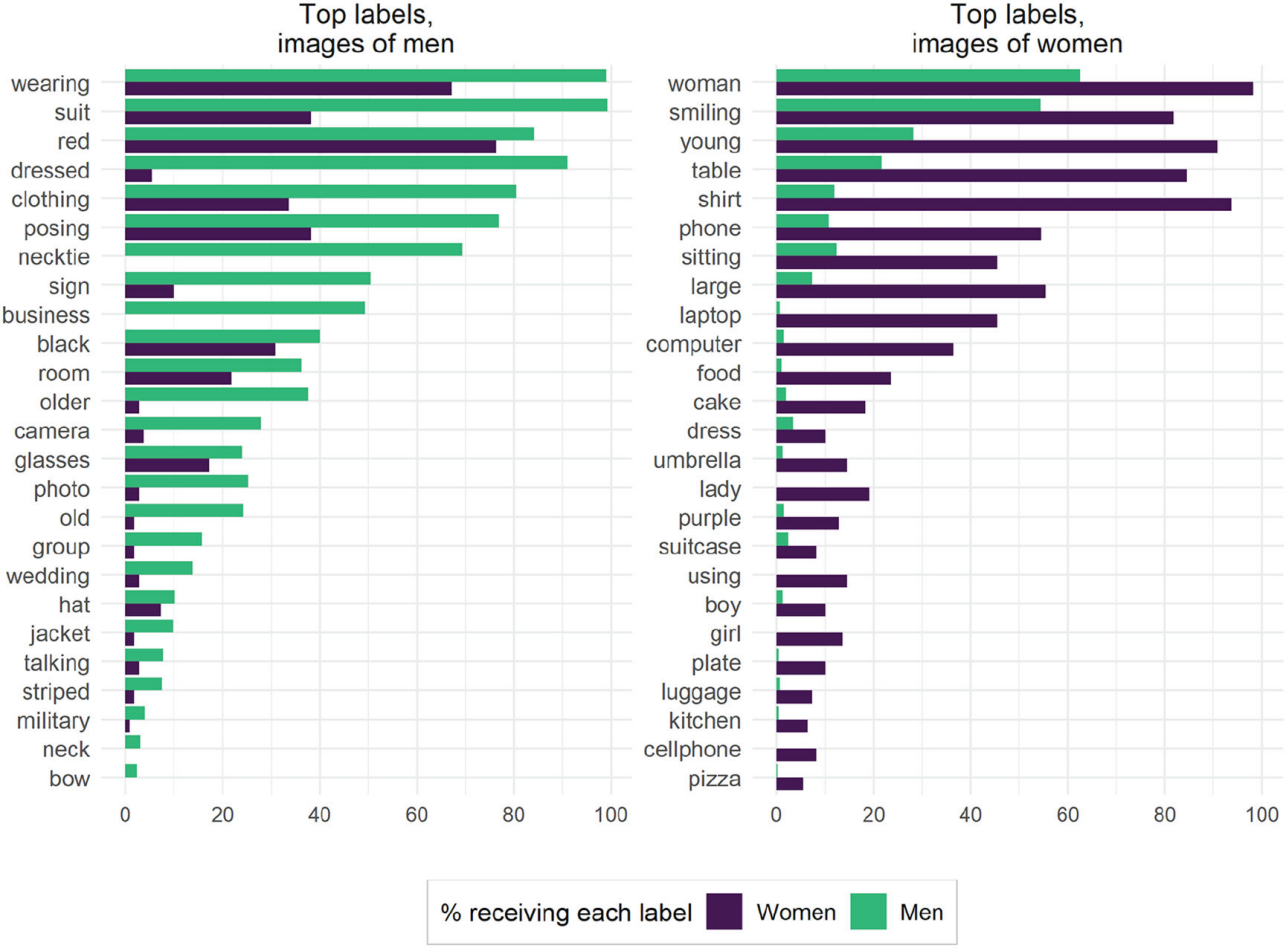
Microsoft Azure Computer Vision labels applied to professional photographs of members of Congress. The 25 most gendered labels for men and women were identified with χ^2^ tests (*p* ≤ .01). Bars denote the percentage of images for a certain label by gender.

Although our results so far have focused on examining gender biases of one particular system, GCV, we also replicated our analysis of our uniform data set of professional photos using two other popular image recognition tools: Amazon Rekognition and Microsoft Azure Computer Vision. We found that labels assigned by these tools produce gender biases similar to GCV (see Figures 9 and 10). For example, Amazon Rekognition assigns the prestigious occupation labels “attorney” and “executive” to photographs of men. Photographs of women are labeled “teen,” “girl,” and “kid,” although the youngest age for both men and women in our data set is 34 years. In addition, images of women are also labeled with “home decor” even when they are from the uniform portrait data set. Unlike GCV and Amazon Rekognition, labels from Microsoft Azure Computer Vision do not seem to be of high precision in general. The system produces biased labels such as “girl,” “cake,” and “kitchen” for portraits of adult women, where no kitchens or food are present. This demonstrates the need for users to evaluate the specific biases of the system they are using at the time they are doing so.

## Discussion

In this article, we have identified systemic and pervasive bias in how images including men and women are processed, such that image recognition systems mimic and even amplify real-world bias. Specifically, we have shown how bias in identification and bias in content skew the results for even uniform political images, labeling photos of women according to their appearance and photos of men according to their occupation. In other words, image-labeling algorithms “see” American congresswomen through the classic gendered stereotypes that have historically beset the visual representation of women, if they see women at all (Goffman 1976; Ferree and Hall 1990). For any project seeking to draw conclusions from labels that image recognition systems apply with a gender bias, gender may further operate as a confounding variable.

Although prior work has sought to either use algorithms (Anastasopoulos et al. 2016; Casas and Webb Williams 2018; Xi et al. 2019) or identify biases in them (Buolamwini and Gebru 2018; Crawford and Paglen 2019; Eubanks 2018), we argue that it is critical for scholars to do both at the same time. Furthermore, we demonstrated that this is different from simply evaluating the correctness of an algorithm’s output, as many applied studies already do. An algorithm such as GCV might be both correct and biased at the same time if it selectively reports a subset of many possible true labels. There is an active field of research focused on constructing algorithms to avoid specific biases (e.g., Kleinberg, Ludwig et al. 2018). But unless algorithms are consciously constructed and tested for that specific purpose, biases are likely to taint applications that rely on their output in unforeseen ways.

Although we have addressed algorithms’ classification of men and women here, it is important to note that a smaller body of work has begun to examine the systematic exclusion of trans and nonbinary people in algorithmic image recognition systems, which relies on conceptions of sex and gender as binary, immutable, and visually legible (Keyes 2018). That is, such algorithms assume that a person or computer can look at someone and know that they are either a man or woman from visual cues such as hairstyle. To be sure, perception by others is a critical dimension of gender and a part of the interactional process of “doing gender” (West and Zimmerman 1987). But because gender is an accomplishment, rather than a presocial fact, observer perception and other dimensions of gender such as individual identity may differ in consequential ways (Lagos 2019). The genders we measure in this article are mostly binary and observer ascribed, either by algorithms or by humans tasked with validating the algorithms. Here, we demonstrate gender biases and stereotypes even within the constrained, binary terms in which the algorithms operate. This complements work on who can be represented in these algorithms by critically evaluating how those who can be represented by a system’s logic are represented by it in practice.

Our findings are necessarily time and context dependent. New training data and model changes will alter these results and may alleviate some of the biases we identified or generate new, unmeasured ones. Nevertheless, research using image-labeling algorithms must be attentive to such biases when drawing conclusions about image content. Our particular results are also specific to the image recognition systems we tested. Among the three systems we evaluated—GCV, Microsoft Azure Computer Vision, and Amazon Rekognition—there was substantial gender bias in every system, but also variation in the specific content and magnitude of biases. Furthermore, the algorithms deployed by Google and other technology companies change frequently. To give one example, GCV has recently removed its gender identification feature from all of its public-facing services (Ghosh 2020).

Furthermore, some kinds of labels we analyzed are not amenable to our bias measurement approach and, we argue, pose substantial measurement reliability challenges. A prominent example of this in our data was the label “smile,” which was applied to women much more often than men in all three commercial image labeling systems we examined. GCV applied the label to congresswomen more than 90 percent of time while applying it to congressmen less than 25 percent of the time. It would be tempting to do analysis of gender bias here: smiling is a highly gendered behavior, particularly in images of women (Goffman 1976). But smiling is far more ambiguous to classify than labels such as “hair,” “outdoors,” “child,” and “military officer.” Researchers who try to create metrics for what counts as a smile invariably find that age, race, gender, nationality, dental health, and more influence not only how people smile but also whether observers see a particular facial expression as a smile (Jensen, Joss, and Lang 1999; Liébart et al. 2004). When one of the authors attempted to tally the presence of smiles in the congressional portraits data, this ambiguity rapidly became apparent: many facial expressions seemed borderline. Was that really a smile? Do smirks count? What if teeth are showing, but they do not seem happy? This is why flight attendants and other emotional laborers are formally trained not just that they are expected to smile, but specifically how they should be smiling (Hochschild 2012). By our count, 91 percent of women and 86 percent of men were smiling—very far from the ratio of smiles in GCV labels and suggestive of substantial gender bias. But our recommendation is that researchers and users should avoid labels with this level of measurement ambiguity altogether.

Beyond simply calling attention to specific, significant gender biases in GCV, this article also serves as a template for future researchers seeking to use commercial algorithms. By comparing biases identified in uniform data sets as well as “found data,” researchers will be better able to evaluate the tools they use before drawing firm conclusions from the data. Although our examples are primarily concerned with gender bias in image labeling, depending on the data set and research question, researchers may use the same procedures to test for bias along any trait and automated labeling system. As our crowdsourced validation suggests that humans predominantly agree with high-confidence labels by GCV, image recognition systems may still be useful for a variety of applications unaffected by gender biases. In any case, we recommend thorough validation efforts before using a commercial image recognition system. To simplify the process of annotating, validating and analyzing images with GCV, one of the investigators of this paper has developed auxiliary open-source software in the form of an R package (Schwemmer 2019).

The increased accessibility of computational tools generally, and computer vision specifically, presents a novel opportunity for social science researchers to expand the study of social life. However, researchers—and practitioners writ large—cannot treat such black-box tools as infallible. With tasks such as image labeling, there are nearly infinite potential labels to describe an image. If “a picture is worth a thousand words,” but an algorithm provides only a handful, the words it chooses are of immense consequence. As some academic disciplines find themselves undergoing a “replication crisis,” reliance on black-box tools that often change without notice can further exacerbate patterns of incorrect inference while even obscuring the methodology used to arrive at these results. As past trends in research methodology in the social sciences have illustrated (Shank 2016), research tools often grow in popularity before their biases and limitations are widely understood. Therefore, our research serves as an injunction to future researchers seeking to break from, rather than reinforce, the biased gendered associations of the past.

## Supplementary Material

online supplement
