# Supplementary material for: Diagnosing Gender Bias in Image Recognition Systems
Source: Socius. Author manuscript; Available in PMC 2022 Aug 4. (PMC9351609; doi:10.1177/2378023120967171)
Supplement: online supplement [file NIHMS1826353-supplement-online_supplement.docx]

**Online Appendix: Diagnosing Gender Bias in Image Recognition Systems**

1. This online appendix includes additional information for our crowdsourced validation, additional notes on methodology as well as results of additional analysis. We relied on a variety of open source tools to conduct our analyses. Replication materials, including the R code used to generate all results shown in the main paper as well as for those included in this supplementary file, are available at [removed for peer review].

**Crowdvalidation Survey**

For human evaluation of image labels, we hired crowd workers on Amazon Mechanical Turk at a rate of $15/hour. The validation survey was reviewed and exempted by Institutional Review Boards at American University (Protocol#: IRB-2020-9), the University of Michigan (HUM00156287), and New York University (IRB-FY2019-3266). From the images tweeted by Members of Congress, we selected a stratified sample of N = 9,250 to conduct the external human validation of GCV labels. *For stratification, we used a weighted randomization strategy. An image’s weight is calculated using both the labels from Google Vision and by the characteristics of the Member of Congress (MC’s) posting the image. Image weights are inversely proportional to how rare their features are, such that images with uncommon labels and coming from MCs from underrepresented groups are more likely to be sampled. Specifically, MCs' images were weighted by the inverse frequency of images from their state, party, house, age, gender, race/ethnicity, and unique ID in the set of all tweets, as well as by the inverse frequency of the labels GCV applied to them. We excluded some labels before sampling. Because the labels “font” and “text” were empirically found to be redundant, we dropped the “font” label. We also dropped 15,631 images that were only labeled “font” and “text,” with no additional labels, as these were the most common images and least informative labels.*

We further excluded images that were thumbnail previews of videos. In our validation data, we only selected labels that GCV assigned ≥ 0.75 confidence to. We presented each worker with 30 images and a set of potential labels for each image. Some labels were assigned by GCV (positive labels); others were chosen at random from the set of GCV labels assigned to other images but not to the one at hand (negative labels). We asked workers to select all labels that applied to each image individually. Each image was coded by at least three workers. An example for one image as included in our validation survey is shown in the following Figure.

**
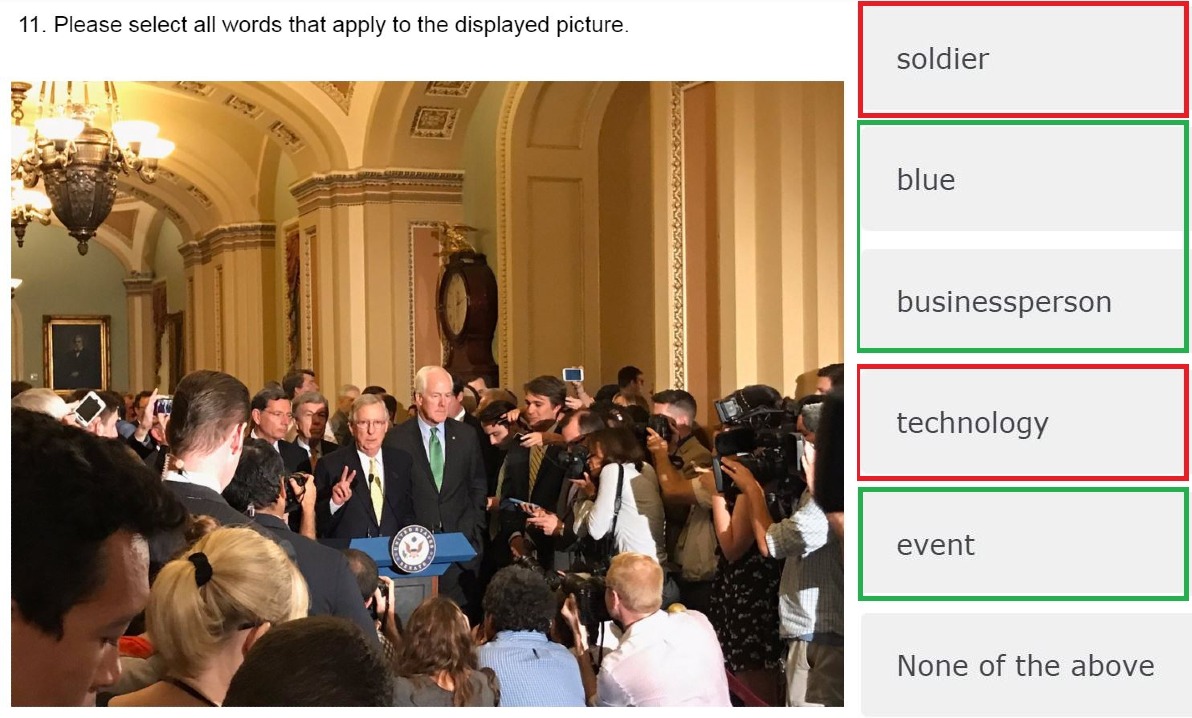
**

**Figure A1.** Example image as shown in our validation survey to crowd workers (without colored margins). Labels with green margins are positive (GCV annotations) and those with red margins are negative (chosen at random from all other labels).

Workers were required to live in the United States. In addition, we required workers to be at least 18 years old. The median age of workers in our sample is 33. Asked what ethnic group best describes themselves, 74% of workers selected “White". Regarding gender, about 60% identified themselves as men, 38% as women and 2% as non-binary, genderqueer, or something else. Workers were also asked with which party they identify: 47% identified themselves as Democrats, 26% as Republicans and 27% either identified themselves with another party or with no party at all. Workers generally agreed with one another. Each person validated the labels of 30 images, and multiple people saw each combination of labels and images. We compute each person’s agreement with the other workers as the fraction of their answers that match other workers’ answers to the same questions. If a worker is guessing at random, we can expect that they will have a label-wise agreement around 0.5, because the selection for each label is a binary choice. Values below 0.5 indicate that a worker disagrees with other workers about labels more often than they agree. Overall, the average agreement is at 0.77 (median=0.79).

Before proceeding with analysis, we excluded responses from 22 people (2.6% of workers) whose agreement with others is below 0.6. We suspect these workers either misunderstood the task or rushed to complete it by randomly guessing (in accordance with the financial incentives of MTurk). Perfect agreement is not expected in this task: many of the labels provided by Google Vision are ambiguous, such as “academic conference” and “tuxedo.” A room full of people with a speaker at the front may look like an academic conference to some workers and a political fundraiser to others. Similarly, some may label any dark suit a “tuxedo,” while others may draw sartorial distinctions between business wear and evening wear.

**Additional notes on methodology**

For identifying the most gendered labels assigned by image recognition software, we rely on two procedures in particular. First, we use $\chi^{2}$ test statistics with Yates correction on labels returned by GCV, Amazon Rekognition and Microsoft Azure Vision. Our test statistics are specified as

$$\chi_{\text{corrected}}^{2}=\sum_{i=1}^{N} \frac{\left( \left| O_{i}-E_{i} \right|-0.5 \right)^{2}}{E_{i}}$$

where $O_{i}$denotes the observed frequency of a label and $E_{i}$ denotes the expected frequency of a label for men or women. Second, we use negative binomial regressions to obtain the expected counts of GCV labels in each coded category for men and women MCs while controlling for ethnicity and party. Regression models are specified as

$$\frac{E\left( y | \boldsymbol{x},x_{k}+\delta\right)}{E\left( y | \boldsymbol{x},x_{k} \right)}=\exp\beta_{k}\delta$$

such that for a change $\delta$ in variable $x_{k}$, the expected count of labels in a category, increases by a factor of $\exp\beta_{k}\delta$, holding all other variables constant (Long and Freese 2006).

**Label categories for Google Cloud Vision**

As explained in the main paper, we further categorized the image labels obtained by GCV for the professional photographs. Three coders, all of whom are authors of this paper, categorized labels into 5 larger categories (see table A1): physical trait / body, occupation, clothing and apparel, color / adjective, and other.

**Table A1**. Image labels as assigned by Google Cloud Vision as well as manually assigned categories.

| ***Label*** | | ***Category*** | ***Label*** | ***Category*** |
| --- | --- | --- | --- | --- |
| *actor* | occupation | | *long_hair* | physical trait / body |
| *afro* | physical trait / body | | *magenta* | color / adjective |
| *bangs* | physical trait / body | | *male* | physical trait / body |
| *beard* | physical trait / body | | *man* | physical trait / body |
| *beauty* | physical trait / body | | *management* | occupation |
| *black_hair* | physical trait / body | | *military_officer* | occupation |
| *blazer* | clothing / apparel | | *military_person* | occupation |
| *blond* | physical trait / body | | *moustache* | physical trait / body |
| *blue* | color / adjective | | *mouth* | physical trait / body |
| *bob_cut* | physical trait / body | | *music_artist* | occupation |
| *brown_hair* | physical trait / body | | *neck* | physical trait / body |
| *business* | occupation | | *necktie* | clothing / apparel |
| *businessperson* | occupation | | *newscaster* | occupation |
| *carpet* | other | | *newsreader* | occupation |
| *cheek* | physical trait / body | | *nose* | physical trait / body |
| *chin* | physical trait / body | | *official* | occupation |
| *dress_shirt* | clothing / apparel | | *outerwear* | clothing / apparel |
| *ear* | physical trait / body | | *pattern* | other |
| *elder* | physical trait / body | | *person* | other |
| *electric_blue* | color / adjective | | *photograph* | other |
| *energy* | other | | *photography* | other |
| *eyebrow* | physical trait / body | | *pink* | color / adjective |
| *eyewear* | clothing / apparel | | *pixie_cut* | physical trait / body |
| *face* | physical trait / body | | *portrait* | other |
| *facial_expression* | physical trait / body | | *portrait_photography* | other |
| *facial_hair* | physical trait / body | | *product* | other |
| *fashion* | clothing / apparel | | *public_speaking* | occupation |
| *fashion_accessory* | clothing / apparel | | *purple* | color / adjective |
| *flag* | other | | *red* | color / adjective |
| *flag_of_the_united_states* | other | | *scarf* | clothing / apparel |
| *forehead* | physical trait / body | | *scholar* | occupation |
| *formal_wear* | clothing / apparel | | *sedan* | other |
| *fun* | color / adjective | | *senior_citizen* | physical trait / body |
| *gentleman* | color / adjective | | *shoulder* | physical trait / body |
| *girl* | physical trait / body | | *skin* | physical trait / body |
| *glasses* | clothing / apparel | | *sleeve* | clothing / apparel |
| *grass* | other | | *smile* | physical trait / body |
| *hair* | physical trait / body | | *speaker* | occupation |
| *hair_coloring* | physical trait / body | | *speech* | other |
| *hairstyle* | physical trait / body | | *spokesperson* | occupation |
| *hat* | clothing / apparel | | *standing* | color / adjective |
| *head* | physical trait / body | | *student* | occupation |
| *headgear* | clothing / apparel | | *suit* | clothing / apparel |
| *hotel_manager* | occupation | | *sunglasses* | clothing / apparel |
| *human* | color / adjective | | *surfer_hair* | physical trait / body |
| *human_hair_color* | physical trait / body | | *sweater* | clothing / apparel |
| *iris* | physical trait / body | | *television_presenter* | occupation |
| *jacket* | clothing / apparel | | *textile* | clothing / apparel |
| *jaw* | physical trait / body | | *tooth* | physical trait / body |
| *jheri_curl* | physical trait / body | | *turquoise* | color / adjective |
| *job* | occupation | | *tuxedo* | clothing / apparel |
| *lady* | physical trait / body | | *uniform* | clothing / apparel |
| *laughter* | physical trait / body | | *vision_care* | clothing / apparel |
| *layered_hair* | physical trait / body | | *white_collar_worker* | occupation |
| *lip* | physical trait / body | |  |  |

Coding reached a satisfactory inter-rater agreement (kappa = 0.878). Our main interest concerns the extent to which men and women are tagged with different label categories as evidence for label bias or its absence. Using the modal category for labels, we then estimated negative binomial count models for each category, predicting the number of labels within that category as a function of the gender, ethnicity, *age,* and party of the portrayed MC. The results are presented in the main text and show that, when estimated on a sample of highly comparable professional photographs, the image labels produced by GCV are strongly gendered. Physical traits / body labels are assigned much more often to images of women MCs, whereas occupation (and to a lesser extent clothing & apparel) are more prevalent among images of men MCs.

We find no such differences for ethnicity, *where we distinguish between “White” and “Non-White” MCs (see the Figure A2).* This may in part stem from the lack of ethnic diversity in the US Congress, as we were only able to compare categories “White" versus “Non-White" due to small number MCs who identify themselves with ethnic groups other than “White".


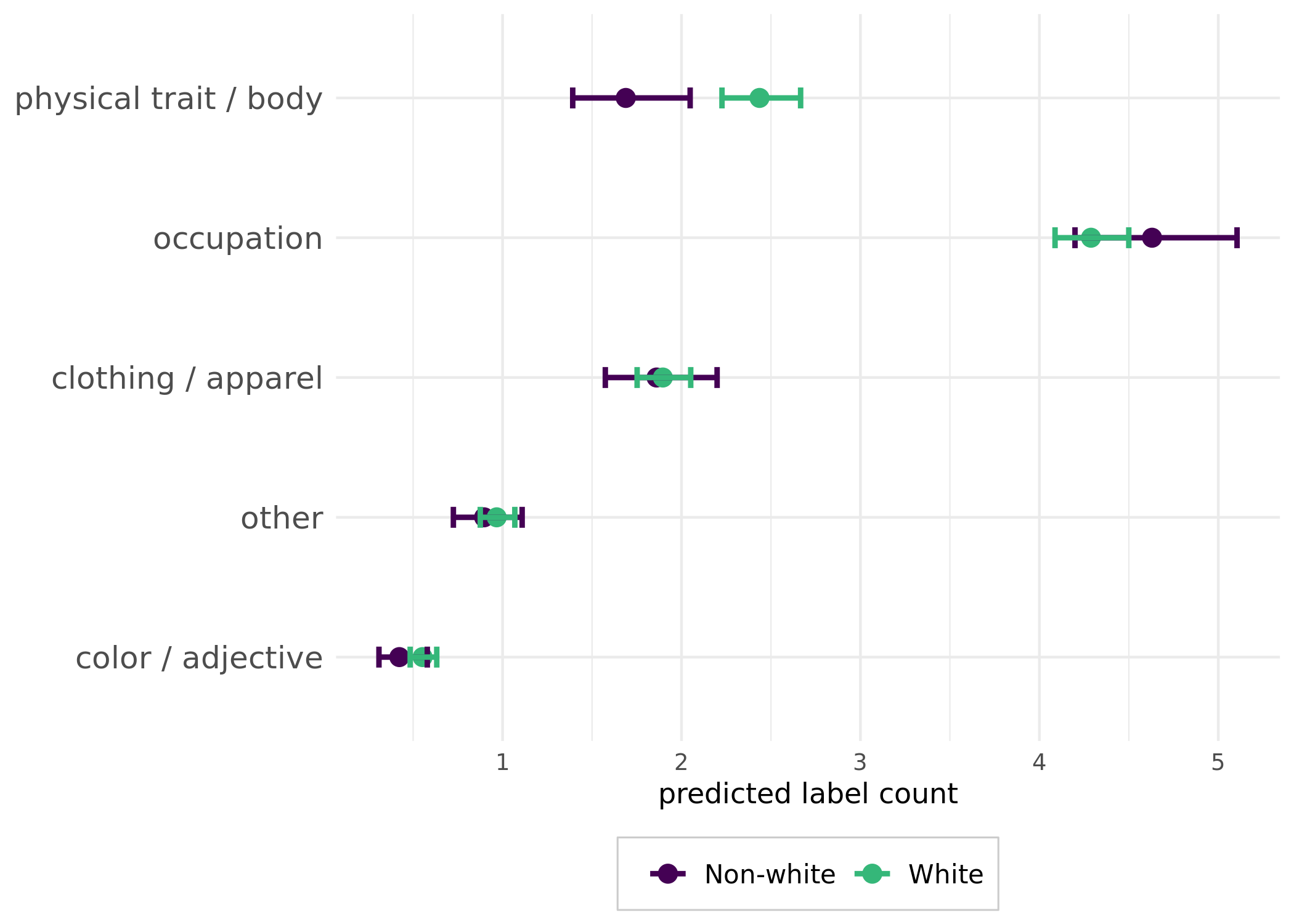


**Figure A2.** Predicted counts of Google Cloud Vision labels in comparison between images of White and Non-white persons. Results are based on photographs of U.S. Members of Congress and negative binomial regressions, controlling for gender, age and party. Circles describe point estimates, bars describe 95% confidence intervals.

Furthermore, we find no substantial differences between Democrats and Republicans and Representatives or Senators for these label categories (see Figure A3).

| 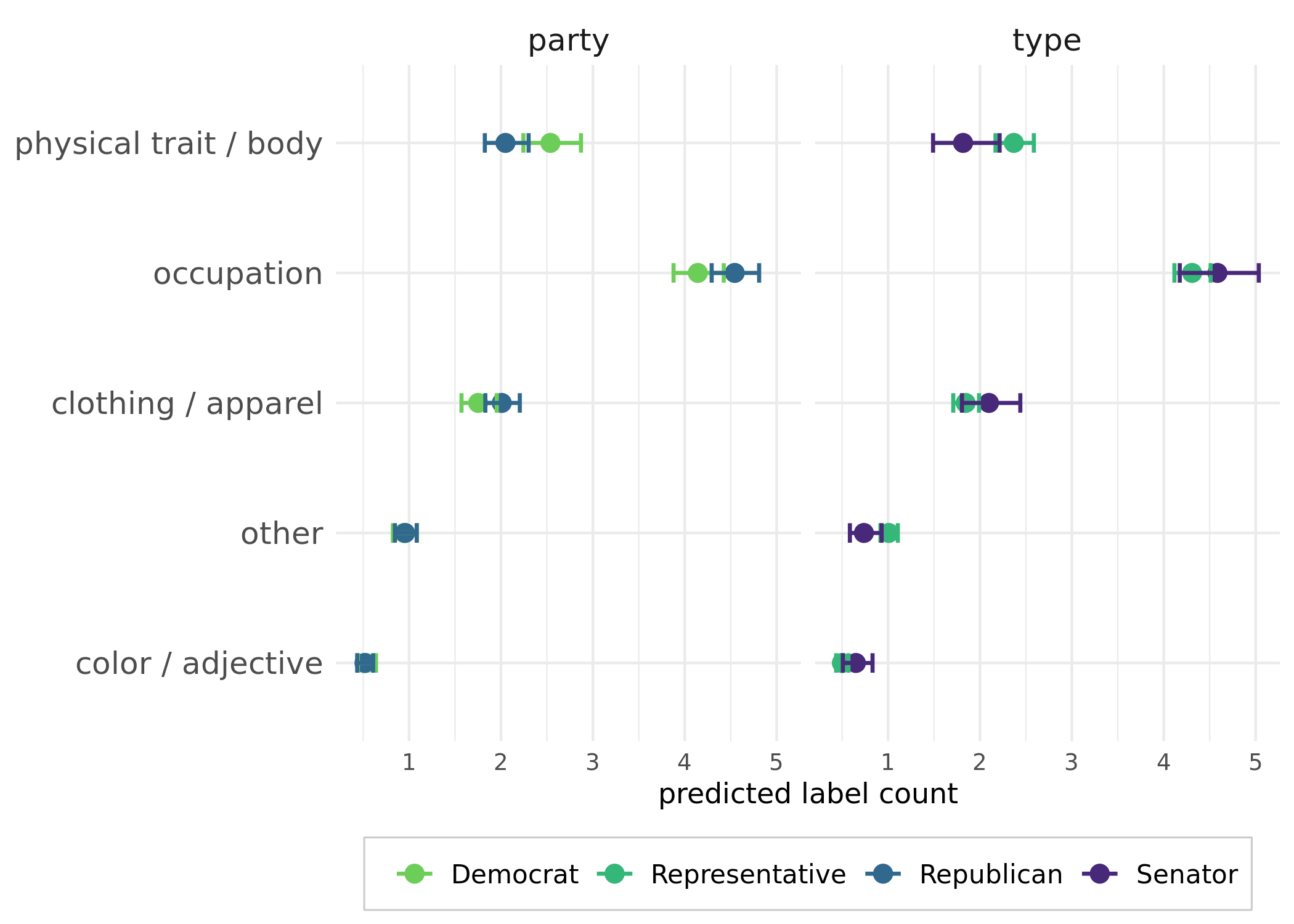 |
| --- |
| **Figure A3.** Predicted counts of Google Cloud Vision labels in comparison between images of Democrats and Republicans, and images of Representatives and Senators. Results are based on photographs of U.S. Members of Congress and negative binomial regressions, controlling for gender, age and ethnicity. Circles describe point estimates, bars describe 95% confidence intervals.  With regards to our control variable age, older Members of Congress were less likely to receive a label related to occupation or clothing & apparel, but more likely to receive labels related to physical traits & body. Women in are dataset are on average older than men (62 vs 60), and the youngest woman and man in our data are both 34 years.  *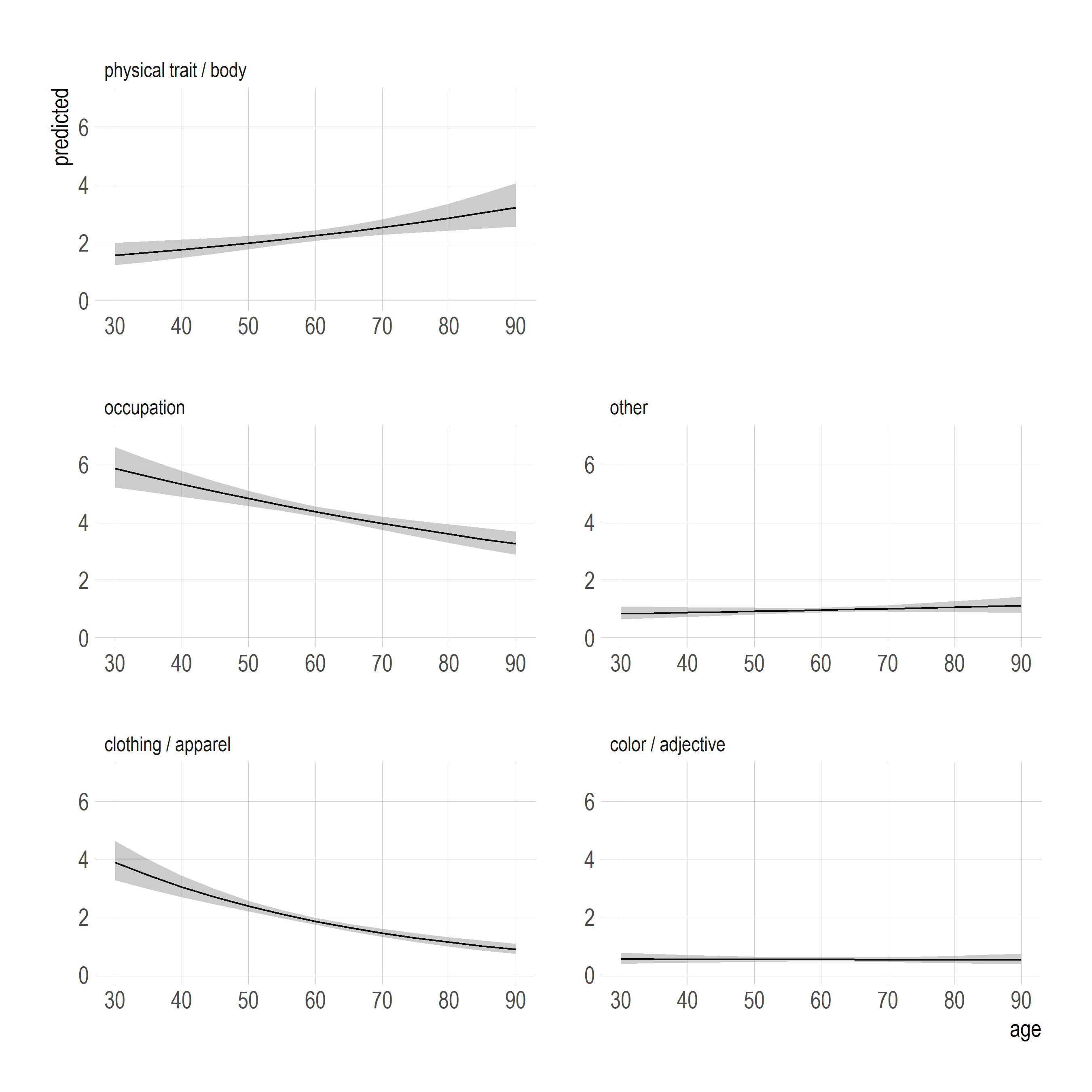*  **Figure A4.** Predicted counts of Google Cloud Vision labels for different age values. Results are based on photographs of U.S. Members of Congress and negative binomial regressions, controlling for gender, party and ethnicity. |

**Label for Twitter images by Party**

In our paper, we show that analyzing the key labels by gender for our Twitter dataset might lead to the wrong conclusion that MCs' gender substantially influences the content of the images they share on Twitter. Since we detect bias in how the Google Cloud Vision platform assigned the labels, we cannot be sure that the differences observed in the image labels are not at least partially confounded by this bias. To examine whether gender differences in labels are similar in our Twitter dataset regardless of MC's partisanship, we again compute $\chi^{2}$ statistics, but this time breaking out the results by both gender and party. For visualization purposes, Figure A5 only displays 20 labels per group without relative frequencies and sorted by $\chi^{2}$ values. These results are similar to those broken out by gender alone. For example, the top label assigned to images tweeted by women in both parties is “girl”, while the top labels for men in both parties are either text-related (e.g. Democratic men) or “official”, “suit”, and “businessperson” (e.g. Republican men). Without detecting biases in the GCV platform, these differences would suggest that MCs' online behavior is different in terms of gender.


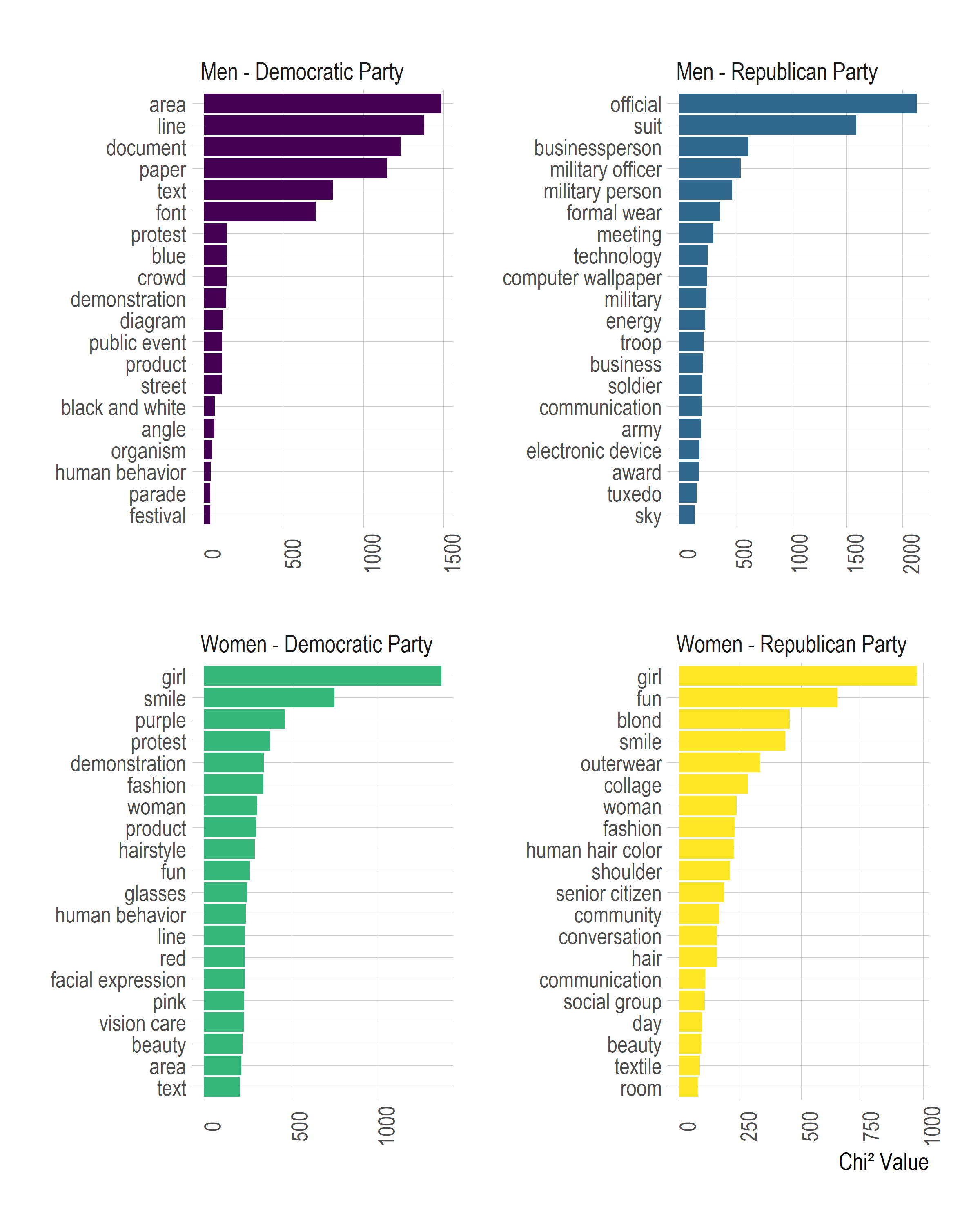


**Figure A5**. Google Cloud Vision labels applied to images tweeted by Members of Congress. The 25 most gendered labels by party and gender were identified with $\chi^{2}$ tests. Bars denote$\chi^{2}$ tests results.

**Image examples for women recognized / not recognized by GCV**

In our paper, we show that the object recognition module of GCV correctly identifies women in images at substantially lower rates in comparison to men. We also qualitatively examined whether any particular features of images could be an indicator for whether men and women are recognized. We did not find any of these features. The following figure shows example of men and women recognized (left-hand column) and not recognized (right-hand column) by GCV.


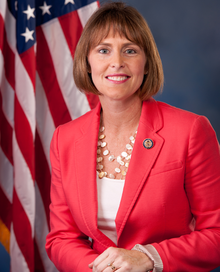

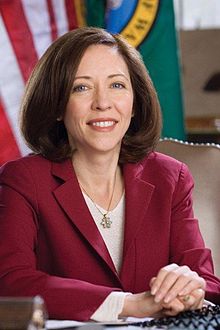


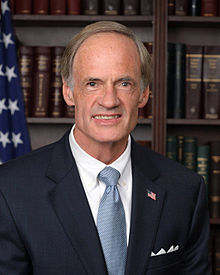


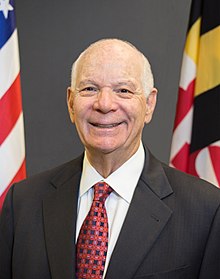


**Figure A6**. Examples for images of men and women recognized / not recognized by Google Cloud Vision’s object recognition. Top-left: Maria Cantwell, recognized. Top-right: Kathy Castor, not recognized. Bottom-left: Ben Cardin, recognized. Bottom-right: Tom Carper, not recognized.
